# Genetic loci for alcohol-related life events and substance-induced affective symptoms: indexing the “dark side” of addiction

**DOI:** 10.1038/s41398-019-0397-6

**Published:** 2019-02-04

**Authors:** Qian Peng, Chris Bizon, Ian R. Gizer, Kirk C. Wilhelmsen, Cindy L. Ehlers

**Affiliations:** 1 Department of Neuroscience, The Scripps Research Institute, La Jolla, CA 92037 USA; 2 Renaissance Computing Institute, University of North Carolina, Chapel Hill, NC 27517 USA; 3 Department of Psychological Sciences, University of Missouri-Columbia, Columbia, MO 65211 USA; 4 Department of Genetics and Neurology, University of North Carolina, Chapel Hill, NC 27599 USA

## Abstract

A limited number of genetic variants have been identified in traditional GWAS as risk or protective factors for alcohol use disorders (AUD) and related phenotypes. We herein report whole-genome association and rare-variant analyses on AUD traits in American Indians (AI) and European Americans (EA). We evaluated 742 AIs and 1711 EAs using low-coverage whole-genome sequencing. Phenotypes included: (1) a metric based on the occurrence of 36 alcohol-related life events that reflect AUD severity; (2) two alcohol-induced affective symptoms that accompany severe AUDs. We identified two new loci for alcohol-related life events with converging evidence from both cohorts: rare variants of K_2P_ channel gene *KCNK2*, and rare missense and splice-site variants in pro-inflammatory mediator gene *PDE4C*. A *NAF1-FSTL5* intergenic variant and an *FSTL5* variant were respectively associated with alcohol-related life events in AI and EA. *PRKG2* of serine/threonine protein kinase family, and rare variants in interleukin subunit gene *EBI3* (*IL-27B*) were uniquely associated with alcohol-induced affective symptoms in AI. LncRNA *LINC02347* on 12q24.32 was uniquely associated with alcohol-induced depression in EA. The top GWAS findings were primarily rare/low-frequency variants in AI, and common variants in EA. Adrenal gland was the most enriched in tissue-specific gene expression analysis for alcohol-related life events, and nucleus accumbens was the most enriched for alcohol-induced affective states in AI. Prefrontal cortex was the most enriched in EA for both traits. These studies suggest that whole-genome sequencing can identify novel, especially uncommon, variants associated with severe AUD phenotypes although the findings may be population specific.

## Introduction

Alcohol use disorders (AUDs) are highly prevalent, disabling disorders that often go untreated in the USA^1^. Although a substantial heritable component has been found to underlie the variation in AUDs (see reviews^2,3^), the identification of specific genetic variants associated with the disorder in genome-wide association studies (GWAS), though appearing promising, has proved to be challenging. The most consistent findings among studies have been variations in alcohol-metabolizing genes alcohol dehydrogenase (*ADH*) and aldehyde dehydrogenase (*ALDH*) that have been shown to confer protection against alcohol dependence in several populations^2,4,5^. In recent years, GWAS have yielded an additional small yet diverse set of single-nucleotide polymorphisms (SNPs) that have been associated with alcohol dependence, alcohol consumption, and related traits in a number of ethnic groups^5–11^. Among these recent findings, β-Klotho (*KLB*) has been repeatedly associated with alcohol consumption in large population studies of European ancestry^11,12^, although alcohol consumption may have significantly different genetic patterns from alcohol use disorders (AUD)^13^. One ethnic group that is particularly understudied yet has a high prevalence of AUD is American Indians (AI)^1,14^. While several studies provide data to demonstrate that a substantial genetic component for risk for AUD exists in the select AI tribes that have been studied^15^, little is known as to the exact genes and genetic variants that may confer this possibly elevated risk, with the exception of the *ADH* and *ALDH* loci^16–18^.

There are several reasons for the paucity of findings linking specific variants to AUDs in any ethnic group. One potential reason may be defining the phenotype. One aspect of AUDs that appears to be highly consistent is the clinical course of the disorder^19,20^. The clinical course, as described by Schuckit et al.^20^, consists of the order and progression of 36 alcohol-related life events. These life events have been shown to be highly similar and consistent across many different subgroups and populations, although age of onset and endorsement rates of individual events can differ^21–29^. Although this phenotype has been extensively described, it has not yet been utilized as a trait to evaluate the genetics of AUDs.

Another phenotype that has been little explored in genetic studies is substance-induced mood disturbance. Among individuals with moderate, and especially severe AUD, mood disturbances can arise that can sometimes mimic major depressive episodes (MDE). This phenomenon was called “secondary depression” and later called substance-induced MDE^30,31^. More subtle substance-induced affective symptoms may also occur during a bout of heavy drinking and/or when an individual cuts down on their drinking or during withdrawal^32^. These more subtle symptoms have been suggested by Koob et al.^33^ as comprising the “dark side” of addiction^33^. It has been further hypothesized that a “negative emotional state” can arise during heavy alcohol exposure that then acts as negative reinforcement that promotes additional drinking in an attempt to eliminate the affective symptoms^34,35^.

Another potential reason for the paucity of genetic findings in AUD is that, similar to other complex diseases, the variants that have so-far been identified in GWAS for AUD-related traits are primarily common variants that may collectively explain only a small portion of the heritability^36^. There are many theories regarding this “missing heritability” of complex diseases^37^. One class of genetic variation that is largely understudied for AUD is rare variants in the genome. Rare variants have been understudied, in part, due to technological constraints limiting comprehensive whole-genome sequencing (WGS) of population samples and the lack of statistical models that incorporate variables such as family relatedness and ethnic admixture. Genotyping followed by imputation to reference panels is insufficient for studying special high-risk populations, as rare variants are often unique to each population. Recent advances in WGS technologies and analytical methods, however, have made possible the identification of both rare and common variants in studies of novel and admixed populations enriched for substance dependence phenotypes, such as American Indians^17,18,38^.

In the present study, we sought to investigate the genetic basis of two understudied phenotypes: (1) the clinical course of AUD as indexed by alcohol-related life events and (2) alcohol-induced affective symptoms, in two independent cohorts: American Indians (AI) and Euro-Americans (EA). Specifically, we conducted: (1) genome-wide association analysis, (2) rare-variant analysis (3) functional and pathway analyses, and (4) tissue-specific gene expression enrichment analysis, using low-coverage whole-genome sequencing data, in order to identify both shared and distinct genetic factors between the two populations.

## Materials and methods

### Participants

Two independent populations were investigated: 742 Native Americans of extended pedigrees from an American Indian cohort (AI), and 1711 primarily Euro-American (EA) participants from the San Francisco Family Alcohol Study (SFFS) (See Table S1 for demographics). We refer to the first cohort as AI and the second as EA or SFFS interchangeably. The population characteristics and the recruitment procedures of the two cohorts have been, respectively, described^39–42^. The protocol of the study of the American Indian cohort was approved by the Scripps Research Institute Institutional Review Board and Indian Health Council, a tribal review group overseeing health issues for the reservations where recruitments took place. The protocol for collection of participants in the SFFS was approved by the University of California San Francisco (UCSF) Committee for the Protection of the Rights of Human Subject while the recruitment took place. Subsequently, the University of North Carolina, at Chapel Hill IRB approved the data analysis plan. Written informed consent was obtained from each participant after study procedures had been fully explained. Participants were compensated for their time spent in the study.

### Phenotypes

The clinical course of AUD as indexed by the 36 alcohol-related life events described by Schuckit et al.^20^ in these two populations has been previously studied^22,23,26^. We herein defined a new weighted alcohol-related life events phenotype: a quantitative trait derived from the 36 alcohol-related life events as listed in Table 1. The life events were given a severity weight of 1 for events 1–12, 2 for 13–24 and 3 for 25–36. The order of the events was based on the mean age of occurrence with the first event happening earliest and the last event (36th) occurring latest in a lifetime. The phenotype was defined as the sum of the severity weights of the 36 alcohol-related life events. The resulting alcohol-related life events for AI and SFFS (EA) cohorts are characterized in Table S2. Of the 1711 individuals in SFFS, 1702 had valid values for this trait. This newly derived phenotype is correlated with DSM5 AUD diagnoses with correlation coefficients ranging from 0.62 to 0.78 and 0.81 for mild to moderate and severe AUDs respectively, in the AIs (Table S3).

**Table 1:** Clinical course of alcohol use disorders: 36 alcohol-related life events, from which the weighted life events phenotype was derived

Severity weight 1	Severity weight 2	Severity weight 3
1. Arguments	13. Binges	25. Arrested for alcohol-related behavior
2. Physical fights	14. Tolerance	26. Problems in love relationship
3. Problems at work/school	15. Interfered with work	27. Considered excessive drinker
4. Problems with family, friends	16. Self injury while drunk	28. Guilt
5. Hitting others without fighting	17. Decreased important activities	29. Wanted to quit 3+ times
6. Objections from family, friends	18. Inability to change drinking behavior	30. Withdrawal
7. Drank while in hazardous situations	19. Morning drinking	31. Unable to quit/cut down
8. Drank when not intended	20. Drank more than intended	32. Arrested for DUI
9. Lost friends	21. Used rules for drinking	33. Shakes
10. Blackouts	22. Little time for non-drinking activities	34. Continues despite health problems
11. Hit/threw things	23. Strong desire for alcohol	35. Health problems occurred
12. Hit family member	24. Psychological impairment	36. Sought professional help

The order of the events was based on the mean age of the occurrence with the first event happening earliest and the last event (36th) occurring latest in a lifetime

Two symptoms, which were assessed across both samples, were used as an index of substance-induced affective states or “dark side symptoms”. The first was a measure of withdrawal that queried whether participants ever felt anxious or depressed when they stopped or cut down on drinking. The second measure queried whether participants’ drinking had ever caused them to feel depressed or uninterested in things for more than 24 h and to the point that it interfered with their functioning. Both phenotypes are dichotomous. The distributions of the two “dark side” phenotypes are also listed in Table S2. These symptoms are rare in moderate and mild AUD and common in severe AUD^32^.

### Whole-genome sequencing and association analysis

The same methods and pipeline were used to conduct low-coverage whole-genome sequencing (LCWGS) on blood-derived DNA for AI and SFFS cohorts, and has been previously published^38^.

A linear mixed model approach as implemented in EMMAX^43^ was used in the whole-genome association analysis, to control for population structures (as AI cohort is primarily admixed)^17,44^ as well as familial relatedness. The association for each variant was conditioned on a kinship matrix that was estimated from the genotypes, in order to capture a wide range of sample structures. We further included sex, age, and age-squared as covariates in all association analyses. The significance of associations was corrected for multiple traits using the effective independent number of traits (*m*_eff_)^45^. For the three traits in the present study, *m*_eff_ = 2.369 and 2.105 for the AI and SFFS cohorts, respectively. We used a *p*-value of 5 × 10^–8^ as the genome-wide significant threshold and 5 × 10^–7^ as the threshold for suggestive significance.

We additionally performed a gene-based test using fastBAT^46^. For each gene, all variants in the range of ± 50 Kb of the gene and of MAF ≥ 1% were included. The *p*-values were corrected for *m*_eff_. The number of genes (*N*) was 24,690 and 24,681 for AI and SFFS, respectively. Thus, the significant threshold for *p*-value was set at 0.05/*N* = 2.0 × 10^–6^, and a suggestive threshold at 2.0 × 10^–5^.

### Gene-based low-frequency and rare variants association analysis

A linear mixed model-based combined multivariate and collapsing method^47^ as implemented in EMMAX was used to collectively analyze the variants having lower than 5% minor allele frequency (MAF). We grouped the low-frequency (1% ≤ MAF < 5%) and rare variants (MAF < 1%) by genes. For each gene, we formed two groups. One group considered all variants on exons, 5′- and 3′-UTRs, upstream and downstream of the gene (denoted as Exon + Reg). The other included only the nonsynonymous and the splicing-site variants (denoted as Nonsyn). Intergenic variants were not considered in the present study. For each variant group type, a gene was excluded if fewer than three markers were found, or if <1% of the samples had any such markers on the gene. The *p*-values were corrected for *m*_eff_. The significant thresholds for corrected *p*-values were set at 0.05/(*N*_Exon+Reg_ + *N*_Nonsyn_) for each trait and cohort, where *N*_Exon + Reg_ is the number of genes in the group Exon + Reg and *N*_Nonsyn_ in the group Nonsyn. Note that correcting for the sum of the numbers of genes in the two groups is likely an overcorrection as two groups of variants are correlated.

### Functional and pathway analyses

Top variants from our association analyses were tested against the brain-specific *cis*-eQTL database BRAINEAC^48^. Polyphen-2 was used to predict whether nonsynonymous variants might be potentially damaging^49^. The variants with *p*-values < 10^–5^ from each GWAS were annotated with genes using SGAdviser^50^; the associated set of genes were then subjected to functional analyses. We used GeneMANIA^51^ to extract potential functional networks, and DAVID 6.8^52^ for disease enrichment analysis.

### Tissue-specific gene expression enrichment analysis

We obtained the median tissue-specific gene expression data from The Genotype-Tissue Expression (GTEx) Project release V7 (at GTEx Portal) for the sets of genes associated with variants that had a *p*-value < 10^–5^ in the GWAS of each trait and cohort. For each gene, its expression profile across tissues was standardized. For each tissue, we then counted the number of genes in each gene set that had expression levels over *z*-score of 2 (representing the most expressed tissues by the gene). If this gene count was significantly higher than expected, the tissue was considered enriched with respect to tissue-specific expressions for the gene set. The significance was determined through permutation tests.

### Data and code availability

The SFFS dataset has been deposited in dbGaP (accession: phs001458.v1.p1). In accordance with the wishes of the tribes no sharing of the AI data are possible. The analysis code is available upon request.

Full details of all the analyses are given in Supplemental Materials and Methods.

## Results

### GWAS for the American Indian cohort

**Fig. 1:**
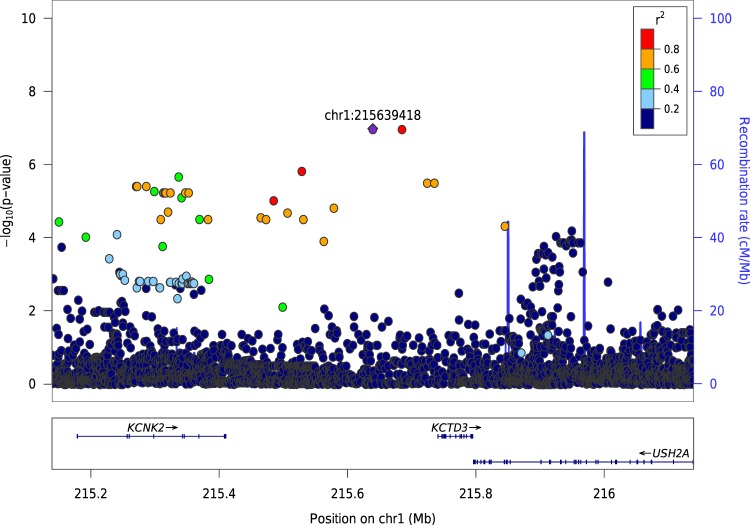
Regional Manhattan plot of *KCNK2* and *KCTD3* variants for alcohol-related life events in AI. Two variants (MAF = 1.4%), upstream of *KCTD3* and downstream of *KCNK2*, were suggestively associated with alcohol-related life events in American Indians (AI) cohort. Gene-based rare variants test also showed that *KCNK2* was associated with alcohol-related life events and affective symptoms when cutting down or during withdrawal. These two single nucleotide polymorphisms (SNPs) are in high LD and also in LD with variants on and near the two nearby genes. They are both near gene activation sites, and located in regions methylated in frontal cortex. Additionally, rs72739250 is located in a CpG island, together suggesting the potential regulatory roles of these two variants^81^

All variants that were found associated with any of the three alcohol-related traits at over a suggestive significant level (*p* < 5 × 10^–7^) in the AI cohort had <5% allele frequency (see Table 2, S5, and Fig. S1). Variant rs200577368, downstream of gene *NAF1* and 658Kbp upstream of *FSTL5* (Fig. S3A), was found significantly associated with alcohol-related life events (*p* = 6.35 × 10^–9^, see Fig. S2A). Variant rs79833306 downstream of *DMRTA1* was also associated with alcohol-related life events (*p* = 5.14 × 10^–8^). Six additional variants were associated with the phenotype at suggestive significant levels (Table 2, S5, and S7), including one SNP located 31Kbp upstream of *PCCA* and 71Kbp downstream of *ZIC2*, and two upstream of gene *KCTD3* and downstream of *KCNK2*, both potassium channel genes (Fig. 1). Additionally, gene-based tests using the fastBAT statistic identified only a single gene, *MME* (a.k.a *NEP*, *CD10*), to be suggestively associated with alcohol-related life events (*p* = 1.47 × 10^–5^).

**Table 2:** Genomic variants for the strongest associations (nominal *p* < 5 × 10^–8^) or the top variant for each AUD-related trait in the American Indian (AI) and the European American (EA) cohorts

Chr	Position	Ref	Alt	dbSNP ID	Genes	Location	*p*-value^a^	*p*-value^b^	Beta	MAF
AI—Alcohol-related life events										
4	163743605	G	T	**rs200577368**	*NAF1-FSTL5*	Intergenic	**2.68E-09**	**6.35E-09**	16.80	0.026
9	22528934	C	T	rs79833306	*DMRTA1*	Downstr	**2.17E-08**	5.14E-08	15.10	0.031
8	41731811	A	C	-	*ANK1*	Intron	**4.44E-08**	1.05E-07	12.59	0.044
13	100709891	C	T	-	*ZIC2-PCCA*	Intergenic	**4.64E-08**	1.10E-07	16.69	0.024
AI—Affective symptoms when cutting down or during withdrawal										
4	82114731	C	T	**rs150351153**	*PRKG2*	Intron	**4.11E-09**	**9.75E-09**	0.62	0.010
7	157162479	A	G	rs139621545	*DNAJB6*	Intron	**4.09E-08**	9.69E-08	0.33	0.031
2	190179895	C	T	rs140545486	*WDR75*	Upstr	**4.35E-08**	1.03E-07	0.51	0.013
AI—24 h of depression when drinking										
3	196296922	A	G	-	*FBXO45*	Intron	**3.61E-08**	8.56E-08	0.80	0.005
15	30281136	T	C	rs75893595	*AK310526*	Upstr	**3.66E-08**	8.68E-08	0.71	0.007
EA—Alcohol-related life events										
4	162501251	T	C	rs11100375	*FSTL5*	Intron	2.29E-07	4.82E-07	-4.25	0.401
EA—Affective symptoms when cutting down or during withdrawal										
6	14778926	C	G	rs2500086	*JARID2*	Upstr	1.34E-07	2.83E-07	-0.09	0.468
EA—24 h of depression when drinking										
12	126920732	A	T	rs4309206	*LINC02347*	Upstr	**4.31E-08**	9.08E-08	-0.09	0.494

See Tables S5, S6 for more comprehensive lists

^a^Nominal *p*-value

^b^Corrected for the effective number of independent traits tested: (*m*_eff_ = 2.369 for AI, 2.105 for EA): *p*-value^a^ × *m*_eff_. Bold font: genome-wide significance

Variant rs150351153, located in an intronic region of *PRKG2*, was significantly associated with affective symptoms during withdrawal in the AI cohort (*p* = 9.75 × 10^–9^, Figs. S2B and S5), and nine others were associated at suggestive significant levels. No variant was significantly associated with alcohol-induced depression. However, ten variants were associated at suggestive significant levels (Fig. S2C).

### GWAS for the European American cohort

No single variant remained genome-wide significant after correcting for the number of traits (Tables 2 and S6, Fig. S2). The top association for alcohol-related life events was rs11100375, a common (MAF = 40%) intronic variant on gene *FSTL5*, at a suggestive significant level (*p* = 4.82 × 10^–7^, Fig. S3B). Note that the top variant rs200577368 associated with the same trait in the AI cohort resides 1.2 Mb upstream of rs11100375 in the intergenic region between *FSTL5* and *NAF1*. Although it’s in linkage disequilibrium (LD) with many variants in the region as reflected by high *D’* (Fig. S3A), there was no clear evidence of LD between rs200577368 and rs11100375. *FSTL5*, a follistatin-like five gene, was most highly expressed in the cerebellar hemisphere. Rs11100375 found *cis*-eQTL for *FSTL5* in the hippocampus, thalamus and substantia nigra regions (FDR = 0.022–0.035).

The top association for alcohol-induced affective symptoms during withdrawal in SFFS was rs2500086 (Fig. S2E). This variant is a *cis*-eQTL for *JARID2* in the substantia nigra (FDR = 5.0 × 10^–4^) and a *cis*-eQTL for *RNF182*, a gene involved in innate immune system, in the frontal cortex (FDR = 5.2 × 10^–4^).

A number of variants in or near a long non-coding RNA *LINC02347* (a.k.a. *LOC100128554*) were associated with alcohol-induced depression at suggestive significance (Figs. S2F and S4). The fastBAT gene-based test also identified this locus as significantly associated with alcohol-induced depression with 155 SNPs included (*p* = 1.77 × 10^–7^). *LINC02347* is located on chromosome 12q24.32, a sub-telomere region. The most significant variant was rs4309206 (*p* = 9.08 × 10^–8^), located upstream of *LINC02347*. Rs4309206 was identified as a *cis*-eQTL for *LINC02347* in occipital cortex (FDR = 0.0024), substantia nigra (FDR = 0.0057), and putamen (FDR = 0.013).

Sequence of *LINC02347* were also partially mapped to transcripts of *FAM32A, CHIA, ROS1, NIPA2*, and *TAP2*. Additionally, top SNPs in high LD with rs4309206 were found to be brain *cis*-eQTLs for *LOC283435*, *LOC400084*, and *TMEM132B* (777Kbp downstream), which are located near *LINC02347* (Fig. S4). For *TMEM132B*, the top eQTLs in brain were rs10847158 (the 2nd most significant SNP) for expression in frontal cortex (FDR = 0.0042), and rs4765395 for expression in white matter (FDR = 0.0023), occipital cortex (FDR = 0.0068), and substantia nigra (FDR = 0.033). All variants that were associated with one of the three AUD-related traits at over a suggestive significant level in the SFFS cohort were common variants (see Tables 2 and S6) and represented *cis*-eQTLs for the related genes in brain regions.

### Gene-based rare-variant analysis for the American Indians

Rare and less-frequent (MAF < 5%) exonic and regulatory (upstream or downstream) variants of a potassium channel gene, *KCNK2*, were significantly associated with alcohol-related life events (*p* = 7.74 × 10^–7^) and suggestively associated with alcohol-induced affective symptoms during withdrawal (*p* = 6.32 × 10^–6^) in the AI cohort (Table 3). The rare coding variants in *KCNK2* were also associated with alcohol-induced affective symptoms during withdrawal in the SFFS cohort at *p* = 0.043 (Table 3). Note that the two variants (MAF = 1.4%) upstream of *KCTD3* that were suggestively associated with alcohol-related life events in AI are also downstream of *KCNK2* (see Fig. 1). *KCNK2* was most highly expressed in fibroblasts and adrenal gland, thyroid and several brain regions. *KCTD3* was more ubiquitously expressed and highly expressed in the adrenal gland.

**Table 3:** Rare and low-frequency variants in genes (MAF < 5%) with the strongest associations in the AI and the EA cohorts

Chr	Positions	Genes^a^	Variants^b^	#Genes^c^	SNPs^d^	% Rare^e^	*p*-value^f^	*p*-value^g^	Beta
AI—Alcohol-related life events									
1	215342573–215410476	**KCNK2**	Exon + Reg	28,112	11(13)	11.59	3.15E-07	**7.47E-07**	10.76
4	16228538–16321722	RP11-783N5.1	Exon + Reg		17(25)	7.82	6.79E-06	1.61E-05	11.25
2	30748587–30863108	LCLAT1	Nonsyn	11,924	3(5)	0.81	1.86E-05	4.41E-05	32.44
19	18321761–18358973	*PDE4C*	Nonsyn		10(11)	5.39	1.24E-03	2.93E-03	-9.65
AI—Affective symptoms when cutting down or during withdrawal									
19	4231323–4237545	**EBI3**	Exon + Reg	28,093	7(10)	3.81	7.05E-08	**1.67E-07**	0.41
1	215342573–215410476	**KCNK2**	Exon + Reg		11(13)	11.70	2.67E-06	**6.32E-06**	0.22
18	28898294–28935292	**DSG1**	Nonsyn	11,901	15(24)	7.35	3.03E-06	**7.17E-06**	0.26
11	47431700–47433920	SLC39A13	Nonsyn		4(5)	3.13	7.84E-05	1.86E-04	0.33
AI—24 h of depression when drinking									
18	73885217–73889608	**RP11–94B19.6**	Exon + Reg	28,093	7(11)	1.63	2.45E-06	**5.81E-06**	0.54
19	4816160–4818488	TICAM1	Exon + Reg		9(15)	6.12	6.64E-06	1.57E-05	0.28
1	91382468–91406787	ZNF644	Nonsyn	11,901	14(14)	7.21	7.82E-06	1.85E-05	0.26
11	124189205–124190036	OR8D2	Nonsyn		4(6)	2.04	2.98E-05	7.07E-05	0.45
EA—Alcohol-related life events									
4	175558059–175750476	GLRA3	Exon + Reg	29,505	91(107)	33.67	4.94E-05	1.04E-04	4.88
19	18321797–18358973	**PDE4C**	Nonsyn	13,717	9(11)	2.29	6.84E-07	**1.44E-06**	19.1
1	215259947–215368442	*KCNK2*	Nonsyn		3(3)	0.71	1.09E-01	2.28E-01	10.9
EA—Affective symptoms when cutting down or during withdrawal									
10	38064292–38073040	RP11-258F22.1	Exon + Reg	29,651	11(12)	7.80	2.52E-05	5.30E-05	-0.19
1	215256673–215410476	*KCNK2*	Exon + Reg		18(22)	15.07	2.07E-02	4.35E-02	0.08
19	2096858–2099250	IZUMO4	Nonsyn	13,900	9(10)	7.86	6.37E-06	1.34E-05	0.20
5	156659347–156675985	ITK	Nonsyn		8(9)	1.77	2.96E-05	6.23E-05	0.37
EA—24 h of depression when drinking									
7	1005685–1015271	COX19	Exon + Reg	29,503	47(59)	29.58	6.53E-06	1.37E-05	-0.11
4	184560761–184580345	RWDD4	Exon + Reg		16(20)	29.05	1.67E-05	3.52E-05	0.11
19	2096858–2099250	IZUMO4	Nonsyn	13,714	9(10)	7.92	1.60E-04	3.36E-04	0.16
19	4231323–4236996	*EBI3*	Nonsyn		3(4)	0.59	4.79E-02	1.01E-01	-0.30

^a^*Bold font*: gene is suggestively significant. *Underlined bold*: The gene is significantly associated with the trait. *Italicized*: replicate in the AI (EA) cohort for a significant gene in the EA (AI) cohort

^b^Exon + Reg: include variants on exon and upstream/downstream of a gene; Nonsyn: include nonsynonymous and splicing variants of a gene.

^c^Number of genes (*N*) included in the test. Genes that have at least three rare/less-frequent markers and %Rare ≥ 1 were included. Significant thresholds were set at 0.05/(*N*_Exon + Reg_ + *N*_Nonsyn_) for each trait in each cohort, using Bonferroni correction. The genome-wide significant threshold was thus set at 1.249E-6, 1.250E-6, 1.250E-6 for the three traits in AI, and 1.157E-6, 1.148E-6, and 1.157E-6 for EA. Note that since the nonsynonymous variants are correlated with exonic variants in each gene, this was likely an overcorrection

^d^Number of markers included in the test. The number in the parenthesis is the total number of markers of the same category on the gene

^e^The fraction of samples that have at least one of the rare/less-frequent markers on the gene

^f^Nominal *p*-values

^g^Corrected with the independent number of effective traits (*m*_eff_ = 2.369 for AI, 2.105 for EA): *p*-value^f^ × *m*_eff_. *Underlined bold font*: genome-wide significance; bold font: suggestive significance

Rare coding variants in *EBI3* (a.k.a *IL27B*) were significantly associated with alcohol-induced affective symptoms during withdrawal (*p* = 1.67 × 10^–7^), followed by *KCNK2*. Only weak support was found for *EBI3* in the Euro-American cohort (nonsynonymous variants in *EBI3* were associated with alcohol-induced depression in SFFS with nominal *p* = 0.048). *EBI3* was most highly expressed in lymphocytes and spleen. Rare, nonsynonymous variants on *DSG1* were suggestively associated with affective symptoms during withdrawal, followed by *SLC39A13. DSG1* was most highly expressed in skin, vagina, and esophagus. Although below the suggestive significant threshold in this rare-variant gene test, *SLC39A13* has recently been identified as a novel locus for alcohol use disorder identification test (AUDIT) total score in the UK Biobank^13^. Rare variants in an lncRNA *RP11-94B19.6* were suggestively associated with alcohol-induced depression, followed by rare coding variants in *TICAM1* (that was most highly expressed in esophagus) and nonsynonymous variants in *ZNF644*.

### Gene-based rare-variant analysis for the Euro-Americans

*PDE4C* was suggestively associated with alcohol-related life events (*p* = 1.44 × 10^–6^) using the rare nonsynonymous or splice-site variants (Table 3). This finding was replicated in the AI cohort (*p* = 2.93 x 10^–3^). Interestingly, although not unexpectedly given that these were rare variants, between the 10 and 9 rare nonsynonymous or splice-site variants, respectively, found in *PDE4C* in the AI and SFFS cohorts, only one variant, rs182916479, at a splice-site was shared across cohorts (0.2% MAF in both cohorts). Of the ten variants in the AI cohort, Polyphen-2 predicted one variant possibly damaging (*probability* = 0.904) and three probably damaging (*prob*. = 0.992–0.999). Of the nine variants in the SFFS cohort, the prediction was one possibly damaging (*prob*. = 0.736) and four probably damaging (*prob*. = 0.986–1).

*IZUMO4* was the top gene for which rare and low-frequency nonsynonymous variants were associated with alcohol-induced affective symptoms during withdrawal. *IZUMO4* was primarily highly expressed in testis. Rare coding and regulatory variants on *COX19* were the top associations for alcohol-induced depression. *COX19* was expressed in many tissues and the most highly expressed in the adrenal gland. None passed suggestive significant threshold after multiple test correction.

Table S7 summarizes relevant functional details of all genes and variants that were significantly or suggestively associated with one of the traits in either cohort.

### Functional analysis and tissue-specific gene expression analysis

The top functional group for alcohol-related life events for the AI cohort was potassium ion transport (FDR = 0.015) (Table S8), while the top functional group for alcohol-induced affective symptoms during withdrawal was with arachidonic acid metabolic process (FDR = 0.068). The top functional groups for alcohol-related life events for the SFFS cohort (Table S9) were regulation of Rac protein signal transduction and regulation of Rac GTPase activity (FDR = 0.11). The top groups for alcohol-induced affective symptoms during withdrawal were response to virus (FDR = 2.64 × 10^–5^) and cellular response to type I interferon (FDR = 7.7 × 10^–4^). No functional group was found significant for alcohol-induced depression for either cohort. The enriched diseases for each trait are listed in Tables S10 and S11 for AI and SFFS, respectively.

**Fig. 2:**
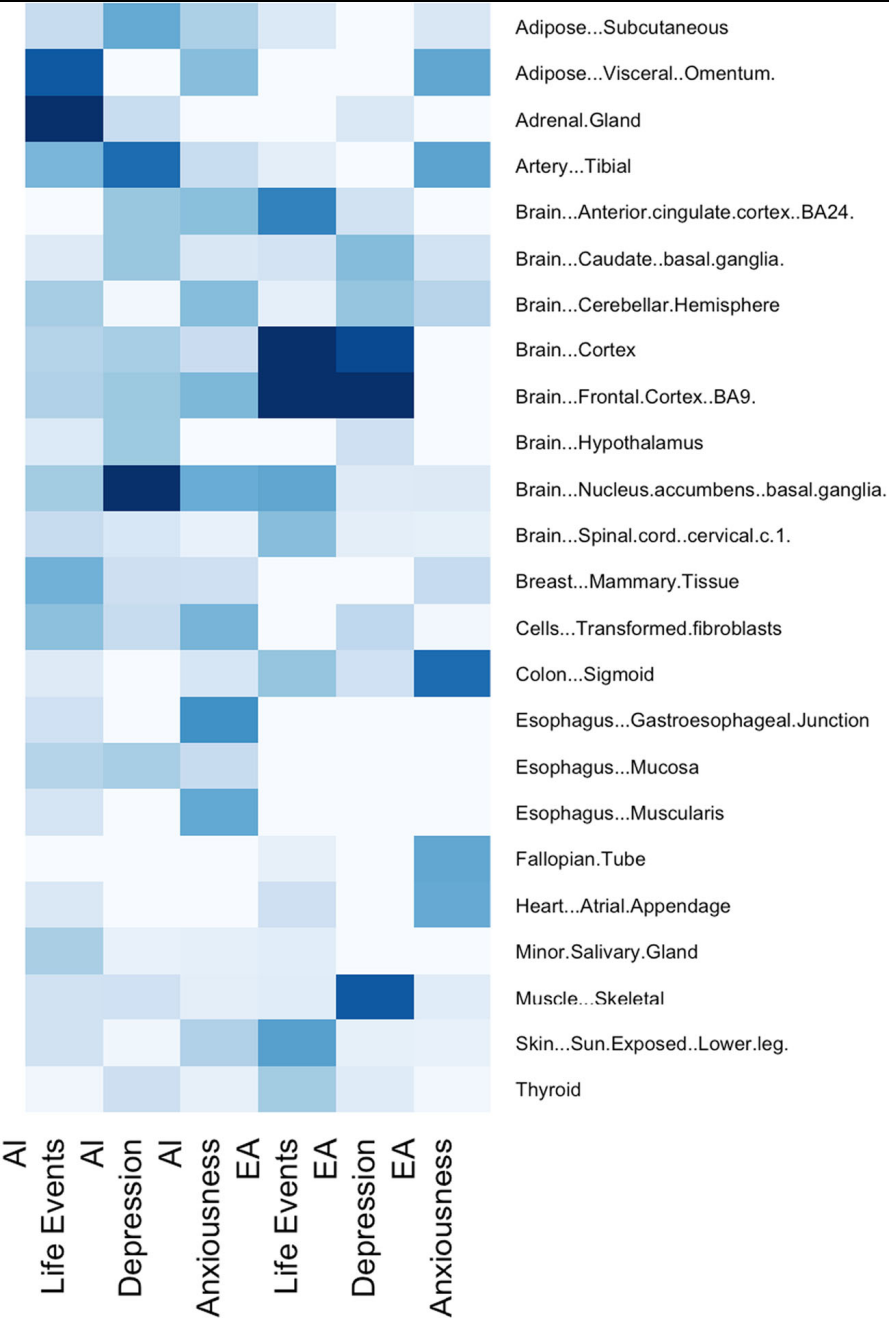
Significance of tissue-specific gene expression enrichment by the top genes associated with one of the three AUD-related traits in the GWAS. Median gene expression by tissues from GTEx V7 was used. Only the most expressed tissues (*z*-score ≥ 2) by a gene were included. Gene sets include those with SNPs that were associated with alcohol-related life events or alcohol-induced affective symptoms in the AI or EA cohort at *p*-value < 10^–5^. Labels: *x*-axis: cohort and trait (Life Events: alcohol-related life events; Depression: 24 h depression when drinking; Anxiousness: affective sympotoms when cutting down or during withdrawal); *y*-axis: GTEx tissue name. The color scale from white to dark-blue corresponds to –log_10_(*p*) = 0–3. Tissues with enrichment *p*-value < 0.1 for all gene sets are omitted. AI American Indians, EA European Americans, SNPs single-nucleotide polymorphisms, GWAS genome-wide association studies

The most enriched tissues with respect to tissue-specific gene expression by the top associated genes were adrenal gland and visceral adipose for alcohol-related life events in AI (see Fig. 2 and Table S12). Esophagus tissues were the most enriched for alcohol-induced affective symptoms during withdrawal, while nucleus accumbens was the most enriched for alcohol-induced depression in AI. In contrast, the most enriched tissues for alcohol-related life events in SFFS were mostly brain tissues including cortex, PFC, anterior cingulate cortex, and nucleus accumbens (Fig. 2 and Table S13). Sigmoid colon was the most enriched for alcohol-induced affective symptoms during withdrawal, followed by the tibial artery and visceral adipose. PFC, cortex, skeletal muscle, and caudate were most enriched for alcohol-induced depression in SFFS.

## Discussion

The present study utilized low-coverage whole-genome sequence data to identify potential variants and pathways underlying two types of phenotypes associated with severe AUD: the severity of the clinical course of AUD and alcohol-induced affective symptoms, in an American Indian and a Euro-American populations.

### Converging evidence from AI and EA suggested two new loci for alcohol-related life events and affective symptoms

Rare variants in a K_2P_ channel gene *KCNK2* were associated with the alcohol-related life events and alcohol-induced affective symptoms during withdrawal in the American Indians. The latter also found some supporting evidence in the Euro-Americans. *KCNK2*, the most studied K_2P_ channel, has been found to play a key role in the cellular mechanisms of neuroprotection, anesthesia, pain-sensing, and depression (see review^53^). It has been shown that *Kcnk2*-knockout mice have increased efficacy of serotonin neurotransmission and are resistant to depression; they also exhibit substantially reduced elevation of corticosterone levels under stress^54^. It has also been shown in humans that *KCNK2* might be related to susceptibility to major depressive disorder (MDD) and involved in antidepressant treatment response^55^. Associated pathways include potassium channels and neuronal system. The Collaborative Study on the Genetics of Alcoholism (COGA) also identified a potassium channel gene *KCNJ6* to be associated with endophenotypes of AUD^56^. Notably, KCNK2 channels can be opened by neuroprotective agents and anesthetics, and inhibited by clinical doses of antidepressant drugs, making it a potential pharmacological target^53^.

Nonsynonymous rare variants in a pro-inflammatory mediator gene, *PDE4C*, were associated with alcohol-related life events in the Euro-American cohort. The association was supported in the AI, although the identified rare variants were largely different. *PDE4C*-encoded protein belongs to the cyclic nucleotide phosphodiesterase (PDE) family and is one of the four PDE4 iso-enzymes, which are the most prevalent PDE in immune cells. The PDE4 inhibitors have long been recognized as anti-inflammatory agents^57^. Preclinical research has found that PDE4 inhibitors reduced ethanol consumption and preference in rodent models by increasing cAMP, thus reducing inflammatory signaling^58–60^. This is consistent with the hypothesis linking neuroimmune signaling with alcohol consumption and dependence^61,62^. Pathways associated with gene *PDE4C* include G protein and GPCR signaling, cAMP signaling, morphine addiction, and opioid signaling^63^. Genes of the same PDE family have previously been implicated in alcohol use. For instance, the COGA has identified a SNP near *PDE11A* to be associated with alcohol dependence^6^. *PDE4B* has been found to be associated with alcohol consumption in the UK Biobank^11^.

Additionally, a low-frequency variant between *NAF1* and *FSTL5* and common variants in *FSTL5* were, respectively, the top variants associated with the alcohol-related life events in the American Indian and the Euro-American cohorts (Fig. S3). Although potentially an interesting locus, there was no clear evidence of LD between the top variants in the two cohorts. Functions of *FSTL5* include calcium ion binding and protein binding. Variants near the gene have been associated with alcohol dependence^64^, response to amphetamines^65^, interferon-γ induced monokine^66^, and paliperidone response in schizophrenia^67^.

### Loci uniquely associated with alcohol-related life events and affective symptoms in the American Indian cohort

A variant in *PRKG2* and rare variants in an interleukin subunit gene *EBI3* (*IL-27B*) were identified for affective symptoms during withdrawal in the AI. *PRKG2* has been associated with obesity traits in a number of ethnic groups^68,69^. The gene has also been associated with EEG alpha power in the COGA cohort^70^. The variant in *PRKG2* is in moderate LD with a few variants in or near *RASGEF1B* (see Fig. S5), a regulator of ICAM-1 in the TLR4/LPS signal transduction pathway involved in pro-inflammatory cytokines release to activate immune response^71^. This gene is ubiquitously expressed in many tissues and has been implicated in MDD^72^. *EBI3* is a subunit of the composite cytokines IL-27 and IL-35. It is involved in IL-27-mediated signaling and cytokine signaling in the immune system and plays a role in cell-mediated immune response. It can also promote pro-inflammatory IL-6 functions by mediating trans-signaling^73^.

### A novel long non-coding RNA was uniquely associated with alcohol-induced depression in the European American cohort

An lncRNA and a gene in chromosome segment 12q24.32 are of particular relevance to alcohol use phenotypes. There has been evidence suggesting that aberrant methylation of *LINC02347* was associated with MDD in European populations^74^. Evidence has also shown that a hemizygous interstitial deletion at chromosome 12q24.31-q24.33 caused multiple dysmorphic features and developmental delay^75^. SNPs in this locus have been shown to act as brain *cis*-eQTLs for *TMEM132B*, which encodes a transmembrane protein in the TMEM132 gene family whose members have been implicated in brain development^76^, panic/anxiety^77^, bipolar disorder^78^, and insomnia^79^. The gene was most highly expressed in tibial nerve and many brain regions, followed by testis and thyroid. *TMEM132B* has been associated with excessive daytime sleepiness (EDS) with BMI adjustment^79^, for which depression was suggested as the most significant risk factor^80^.

In summary, this study presents the first genome-wide analysis of an AUD clinical course severity phenotype and alcohol-induced affective symptoms (“dark side” traits) in two independent populations: American Indians and Euro-Americans. We have identified several novel loci containing rare variants. Many associated genes show increased expression in brain regions, adrenal gland, and digestive track, confirming the importance of neuronal, stress, immune, and metabolic systems in AUDs. However, certain limitations should be considered when making inferences from these findings. At the moderate sample sizes of 742 and 1711 of the AI and EA cohorts, respectively, we had limited statistical power to detect genome-wide significant associations (see Fig. S6 for power calculations for the study). Further, given the uniqueness of our American Indian sample, there are presently no replication samples available for the LCWGS study in AI, although there was corroborative evidence between the AI and the EA cohorts (two independent populations) to support two of the top rare-variant gene findings. Although all of the top GWAS variants from the EA cohort were found to be *cis*-eQTLs for certain brain regions, there was no eQTL information available in the public domain for any of the AI top variants, likely because they were all low-frequency variants. The fact that low-frequency variants predominated our findings, especially for the AI cohort, suggests that rare and less-frequent variants may play important roles in complex diseases such as AUD, especially in unique high-risk populations such as American Indians.

## Supplementary information

Supplemental Files
