# Supplementary material for: Genetic loci for alcohol-related life events and substance-induced affective symptoms: indexing the “dark side” of addiction
Source: Transl Psychiatry. 2019 Feb 4;9:71. doi: 10.1038/s41398-019-0397-6 (PMC6362044; doi:10.1038/s41398-019-0397-6)
Supplement: Supplementary file 1 — Supplemental Files [file 41398_2019_397_MOESM1_ESM.docx]

Table of Contents

Supplemental Figures 2

Figure S1. Q-Q plots of GWAS 2

Figure S2. Manhattan plots of GWAS for AUD traits 3

Figure S3. LocusZoom plot of top variants for alcohol-related life events 4

Figure S4. LocusZoom plot of top variants for alcohol-induced depression in EA 5

Figure S5. LocusZoom plot of *PRKG2* variant for alcohol-induced affective symptoms in AI 6

Figure S6. Statistical powers 7

Supplemental Tables 8

Table S1. Demographics of the two population samples. 8

Table S2. Statistical characteristics of the phenotypes 9

Table S3. Correlations between studied traits and DSM5 AUDs 10

Table S4. Phenotype correlation matrix 10

Table S5. GWAS results for AI 11

Table S6. GWAS results for EA 13

Table S7. Comprehensive list of loci and relevant functions associated with the AUD traits in AI and/or EA 15

Table S8. Top functional groups in AI 21

Table S9. Top functional groups in EA 22

Table S10. Disease enrichment in AI 23

Table S11. Disease enrichment in EA 24

Table S12. Tissue-specific gene expression enrichment in AI 25

Table S13. Tissue-specific gene expression enrichment in EA 26

Supplemental Materials and Methods 27

Participants 27

Whole genome sequencing and variants calling 27

Whole genome association analysis 28

Effective number of independent phenotypes 30

Gene-based low-frequency and rare variants association analysis 30

Functional and pathway analyses 31

Tissue-specific gene expression enrichment analysis 32

Supplemental References 34

# Supplemental Figures

## Figure S1. Q-Q plots of GWAS

Q-Q plots of GWAS for AUD-related traits in AI (A-C) and EA (D-F).

## Figure S2. Manhattan plots of GWAS for AUD traits

Panels A, B, C: American Indian cohort; Panels D, E, F: European American cohort.

##
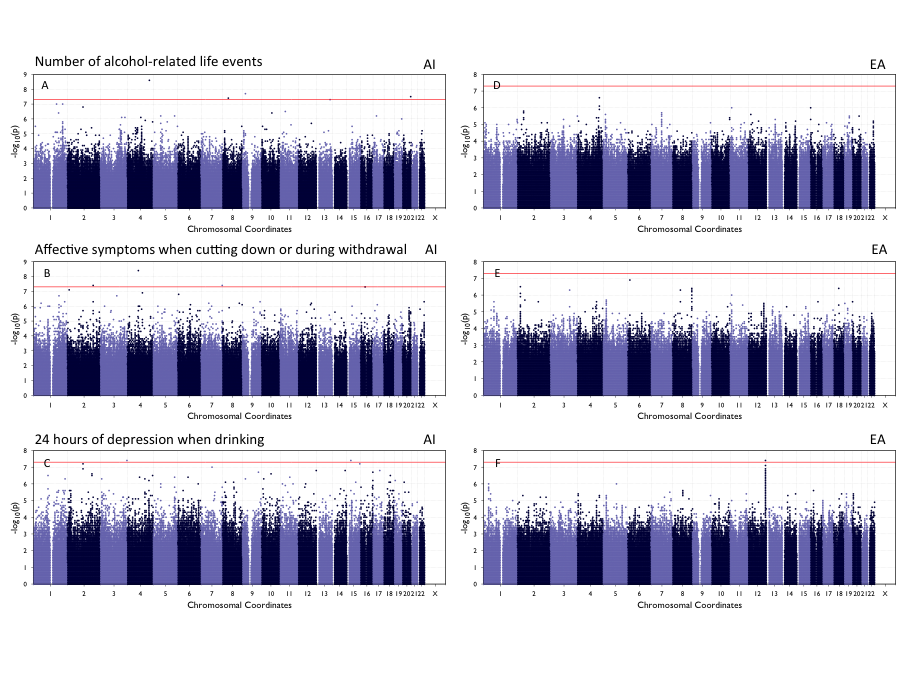


## Figure S3. LocusZoom plot of top variants for alcohol-related life events

Top variants associated with alcohol-related life events in AI (panel A) and SFFS (panel B) are in nearby genomic region. LD measure in figure A is *D’* (top SNP has 2.6% MAF thus low *r^2^* (<0.2) with other SNPs), and *r^2^* in figure B.

## Figure S4. LocusZoom plot of top variants for alcohol-induced depression in EA

Variants on or near long intergenic noncoding RNA *LINC02347* (*LOC100128554*) were associated with alcohol-induced depression (24 hours of depression when drinking) in SFFS cohort.

## Figure S5. LocusZoom plot of *PRKG2* variant for alcohol-induced affective symptoms in AI

Variant rs150351153 (MAF=1%) on gene *PRKG2* was significantly associated with alcohol-induced affective symptoms when cutting down or during withdrawal in the AI cohort.

## Figure S6. Statistical powers

A, B: Statistical power as a function of variance explained by the marker (effect size) to detect genomic association with sample size *n* = 742, 1711. C: Desired sample size to detect association for given effect sizes in order to achieve 80% statistical power. The significant level or type I error rate is set at 5×10^−8^ (typical GWAS significant threshold) and 5×10^−7^ (suggestive significance). The power calculation was done using the Genetic Power Calculator ^1^. Only additive effects were considered. The samples were assumed unrelated. The relatedness in the samples generally yield slightly increased powers ^2^.

# Supplemental Tables

## Table S1. Demographics of the two population samples.

| Cohort | American Indian  (AI) | UCSF Family Alcohol Study (EA/SFFS) |
| --- | --- | --- |
| Samples | 742 | 1711 |
| Family | 170 | 807 |
| Age^1^ (yrs) | 31.2±13.2 [18, 82] | 49.4+/-13.0 [17,84] |
| Gender | M: 317, F: 425 | M: 650, F: 1061 |
| Ethnicity | Native American | European American 95%  African American 3%  Native American 1%  Others 1% |

^1^ In the format of mean±s.d. [min, max]

## Table S2. Statistical characteristics of the phenotypes

Statistical characteristics of alcohol-related life events and alcohol-induced affective symptoms (dark side traits). No further transformation was applied to the phenotypes to avoid potentially invalid inferences. Note that linear regression, as in the association analysis for quantitative traits, does not require normal distribution of the dependent variable (or residuals) for sufficiently large samples where Central Limit Theorem applies ^3^.

| **Cohort** | **AI** | | **SFFS/EA** | |
| --- | --- | --- | --- | --- |
| Total Samples | 742 | | 1711 | |
| Alcohol-related life events | *N* = 742 | | *N* = 1702 | |
| Range | 0 - 69 | | 0 - 72 | |
| Median | 18 | | 35 | |
| Mean | 22.60 | | 30.53 | |
| S.D. | 18.81 | | 24.49 | |
| Skewness | 0.62 | | -0.06 | |
| Kurtosis | 2.27 | | 1.48 | |
| Dark-side traits | No | Yes | No | Yes |
| Affective symptoms when cutting down or during withdrawal | 584 | 151 | 986 | 706 |
| 24hr Depression when drinking | 591 | 144 | 1064 | 640 |

## Table S3. Correlations between studied traits and DSM5 AUDs

Alcohol related life events and AUD dark side traits are highly correlated with severe DSM5 AUD in AI. Correlations were taken between traits with sex, age and age-squared as covariates.

|  | Life Events | Affective Symptoms | 24hr Depression |
| --- | --- | --- | --- |
| Affective Symptoms | 0.615 |  |  |
| 24hr Depression | 0.581 | 0.480 |  |
| DSM5 AUD Mild | 0.624 | 0.281 | 0.294 |
| DSM5 AUD Moderate | 0.775 | 0.436 | 0.408 |
| DSM5 AUD Severe | 0.807 | 0.524 | 0.483 |

## Table S4. Phenotype correlation matrix

Phenotypes are correlated. Correlations were taken between traits with sex, age and age-squared as covariates. The effective numbers of independent traits derived from the correlation matrix are 2.369 and 2.105 for AI and SFFS/EA respectively.

| SFFS\AI^1^ | Life Events | Affective Symptoms^2^ | 24hr Depression^3^ |
| --- | --- | --- | --- |
| Life Events |  | 0.615 | 0.581 |
| Affective Symptoms | 0.752 |  | 0.480 |
| 24hr Depression | 0.684 | 0.556 |  |

^1^ The lower triangle is for SFFS, and upper triangle is for AI.

^2^ Affective symptoms when cutting down or during withdrawal.

^3^ 24 hours of depression when drinking.

## Table S5. GWAS results for AI

Genomic variants for the strongest associations (nominal *p* < 5e-7) for the American Indian cohort

| CHR | POS | Ref | Alt | dbSNP ID | Gene | Location | P-value^1^ | P-value^2^ | Beta | MAF |
| --- | --- | --- | --- | --- | --- | --- | --- | --- | --- | --- |
| **Alcohol-related life events** | | | | | | | | | | |
| 4 | 163743605 | G | T | **rs200577368** | NAF1-FSTL5 | Intergenic | 2.68E-09 | **6.35E-09** | 16.80 | 0.026 |
| 9 | 22528934 | C | T | rs79833306 | DMRTA1 | Downstr | 2.17E-08 | **5.14E-08** | 15.10 | 0.031 |
| 8 | 41731811 | A | C | - | ANK1 | Intron | 4.44E-08 | **1.05E-07** | 12.59 | 0.044 |
| 13 | 100709891 | C | T | - | ZIC2-PCCA | Intergenic | 4.64E-08 | **1.10E-07** | 16.69 | 0.024 |
| 1 | 169344460 | C | T | - | BLZF1 | Intron | 9.42E-08 | **2.23E-07** | 13.34 | 0.035 |
| 1 | 215639418 | A | T | rs145751732 | KCTD3 | Upstr | 1.05E-07 | **2.48E-07** | 21.12 | 0.014 |
| 1 | 215684926 | G | A | rs72739250 | KCTD3 | Upstr | 1.05E-07 | **2.48E-07** | 21.12 | 0.014 |
| 2 | 114762469 | G | T | - | CR601322 | Intron | 1.51E-07 | **3.58E-07** | 21.64 | 0.013 |
| 11 | 40279173 | T | C | - | LRRC4C | Intron | 3.34E-07 | 7.90E-07 | 23.40 | 0.010 |
| 10 | 75475555 | T | G | - | AGAP5 | Intron | 4.16E-07 | 9.85E-07 | 24.89 | 0.009 |
| 1 | 185988429 | G | T | - | HMCN1 | Intron | 4.27E-07 | 1.01E-06 | 18.75 | 0.016 |
| **Affective symptoms when cutting down or during withdrawal** | | | | | | | | | | |
| 4 | 82114731 | C | T | **rs150351153** | PRKG2 | Intron | 4.11E-09 | **9.75E-09** | 0.62 | 0.010 |
| 7 | 157162479 | A | G | rs139621545 | DNAJB6 | Intron | 4.09E-08 | **9.69E-08** | 0.33 | 0.031 |
| 2 | 190179895 | C | T | rs140545486 | WDR75 | Upstr | 4.35E-08 | **1.03E-07** | 0.51 | 0.013 |
| 16 | 32358728 | A | G | rs4099387 | DQ578965 | Upstr | 5.22E-08 | **1.24E-07** | 0.72 | 0.006 |
| 2 | 13095692 | T | G | rs72769625 | AK123120 | Downstr | 8.02E-08 | **1.90E-07** | 0.62 | 0.008 |
| 4 | 112423234 | A | G | rs76397899 | C4orf32 | Upstr | 1.25E-07 | **2.95E-07** | 0.41 | 0.019 |
| 4 | 112475916 | T | C | rs146300984 | C4orf32 | Upstr | 1.25E-07 | **2.95E-07** | 0.41 | 0.019 |
| 6 | 7175729 | G | A | rs115143840 | RREB1 | Intron | 1.54E-07 | **3.64E-07** | 0.57 | 0.010 |
| 1 | 187484040 | T | A | rs114401008 | C1orf99 | Upstr | 2.07E-07 | **4.91E-07** | 0.51 | 0.012 |
| 1 | 187510635 | T | C | rs142150804 | C1orf99 | Upstr | 2.07E-07 | **4.91E-07** | 0.51 | 0.012 |
| 3 | 122300818 | G | T | rs183811860 | PARP15 | Intron | 2.12E-07 | 5.03E-07 | 0.56 | 0.008 |
| 3 | 122304319 | G | A | rs184658435 | PARP15 | Intron | 2.12E-07 | 5.03E-07 | 0.56 | 0.008 |
| 22 | 49355859 | A | T | rs113838057 | BC037788 | Downstr | 4.56E-07 | 1.08E-06 | 0.25 | 0.046 |
| 1 | 229129788 | G | A | rs1417969 | AX748369 | Upstr | 4.66E-07 | 1.10E-06 | 0.13 | 0.203 |
| **24 hours of depression when drinking** | | | | | | | | | | |
| 3 | 196296922 | A | G | - | FBXO45 | Intron | 3.61E-08 | **8.56E-08** | 0.80 | 0.005 |
| 15 | 30281136 | T | C | rs75893595 | AK310526 | Upstr | 3.66E-08 | **8.68E-08** | 0.71 | 0.007 |
| 15 | 95883433 | G | A | - | BC040875 | Upstr | 6.17E-08 | **1.46E-07** | 0.76 | 0.005 |
| 2 | 116314604 | G | A | rs10189544 | DPP10 | Intron | 6.56E-08 | **1.56E-07** | -0.74 | 0.006 |
| 7 | 82761185 | T | A | rs144506483 | PCLO | Intron | 9.08E-08 | **2.15E-07** | 0.77 | 0.005 |
| 2 | 115558696 | C | T | - | DPP10 | Intron | 1.28E-07 | **3.03E-07** | 0.73 | 0.006 |
| 14 | 97964780 | C | T | - | BC038465 | Downstr | 1.45E-07 | **3.42E-07** | 0.77 | 0.005 |
| 17 | 52323367 | G | A | - | KIF2B | Downstr | 1.46E-07 | **3.45E-07** | 0.34 | 0.027 |
| 12 | 131864446 | C | T | rs117113518 | LOC338797 | Downstr | 1.46E-07 | **3.45E-07** | 0.67 | 0.007 |
| 9 | 117198179 | T | A | - | DFNB31 | Intron | 1.83E-07 | **4.34E-07** | 0.27 | 0.042 |
| 16 | 89520316 | G | A | rs139559418 | ANKRD11 | Intron | 2.14E-07 | 5.07E-07 | 0.45 | 0.016 |
| 2 | 181999799 | T | A | rs114210617 | AK125001 | Intron | 2.31E-07 | 5.48E-07 | 0.38 | 0.022 |
| 10 | 71485170 | A | C | rs4990193 | COL13A1 | Upstr | 2.39E-07 | 5.67E-07 | 0.15 | 0.183 |
| 1 | 107471775 | C | T | rs140029904 | PRMT6 | Upstr | 2.86E-07 | 6.77E-07 | 0.58 | 0.010 |
| 18 | 49303210 | C | T | rs187181120 | AK294844 | Downstr | 2.91E-07 | 6.90E-07 | 0.43 | 0.017 |
| 4 | 186634332 | T | C | - | SORBS2 | Intron | 3.14E-07 | 7.44E-07 | 0.47 | 0.011 |
| 2 | 181982005 | A | G | rs116222527 | AK125001 | Upstr | 3.15E-07 | 7.46E-07 | 0.39 | 0.021 |
| 5 | 157954078 | T | C | rs78188191 | EBF1 | Downstr | 3.61E-07 | 8.54E-07 | 0.50 | 0.012 |
| 5 | 157959266 | A | G | rs17702646 | EBF1 | Downstr | 3.61E-07 | 8.54E-07 | 0.50 | 0.012 |
| 11 | 72933951 | G | A | rs73536982 | P2RY2 | Intron | 3.61E-07 | 8.56E-07 | 0.38 | 0.022 |
| 4 | 89783557 | G | A | rs56403871 | FAM13A | Intron | 3.69E-07 | 8.74E-07 | 0.42 | 0.017 |
| 6 | 77257134 | C | G | rs193278576 | IMPG1 | Upstr | 4.07E-07 | 9.65E-07 | 0.19 | 0.097 |
| 10 | 17364239 | G | A | rs10607193 | ST8SIA6 | Intron | 4.49E-07 | 1.06E-06 | 0.21 | 0.063 |
| 1 | 231116454 | C | T | rs144811688 | ARV1 | Intron | 4.50E-07 | 1.07E-06 | 0.51 | 0.012 |
| 4 | 130488015 | T | C | rs72673937 | BC035172 | Downstr | 4.60E-07 | 1.09E-06 | 0.71 | 0.006 |

^1^ Nominal *p*-value.

^2^ Corrected for the effective number of independent traits tested: *p*-value^1^x*m_eff_*, and *m_eff_* = 2.369 for AI. Underlined bold font: genome-wide significance (corrected *p*<5E-8); bold font: suggestive significance (corrected *p*<5E-7).

## Table S6. GWAS results for EA

Genomic variants for the strongest associations (nominal *p* < 5e-7) for the European American (EA/SFFS) cohort

| CHR | POS | Ref | Alt | dbSNP ID | Gene | Location | P-value^1^ | P-value^2^ | Beta | MAF |
| --- | --- | --- | --- | --- | --- | --- | --- | --- | --- | --- |
| **Alcohol-related life events** | | | | | | | | | | |
| 4 | 162501251 | T | C | rs11100375 | FSTL5 | Intron | 2.29E-07 | **4.82E-07** | -4.25 | 0.401 |
| **Affective symptoms when cutting down or during withdrawal** | | | | | | | | | | |
| 6 | 14778926 | C | G | rs2500086 | JARID2 | Upstr | 1.34E-07 | **2.83E-07** | -0.09 | 0.468 |
| 2 | 21595204 | A | C | rs219521 | AK090620 | Downstr | 3.15E-07 | 6.63E-07 | 0.09 | 0.336 |
| 2 | 21592362 | G | C | rs219516 | AK090620 | Downstr | 3.35E-07 | 7.06E-07 | 0.09 | 0.348 |
| 8 | 142154291 | G | A | rs307761 | DENND3 | Exon | 3.74E-07 | 7.87E-07 | 0.13 | 0.118 |
| 8 | 142160876 | C | T | rs170088 | DENND3 | Intron | 3.95E-07 | 8.31E-07 | 0.13 | 0.119 |
| 18 | 35979462 | C | T | rs193051512 | LOC647946 | Downstr | 4.40E-07 | 9.25E-07 | 0.32 | 0.018 |
| 8 | 142157260 | T | C | rs307758 | DENND3 | Intron | 4.99E-07 | 1.05E-06 | 0.13 | 0.119 |
| **24 hours of depression when drinking** | | | | | | | | | | |
| 12 | 126920732 | A | T | rs4309206 | LINC02347 | Upstr | **4.31E-08** | **9.08E-08** | -0.09 | 0.494 |
| 12 | 126938862 | G | A | rs10847158 | LINC02347 | Intron | 7.33E-08 | **1.54E-07** | -0.09 | 0.487 |
| 12 | 126930567 | C | T | rs7296549 | LINC02347 | Intron | 8.08E-08 | **1.70E-07** | -0.09 | 0.494 |
| 12 | 126930439 | T | C | rs7315379 | LINC02347 | Intron | 8.85E-08 | **1.86E-07** | -0.09 | 0.496 |
| 12 | 126926620 | C | G | rs10847153 | LINC02347 | Upstr | 1.29E-07 | **2.72E-07** | -0.09 | 0.493 |
| 12 | 126924513 | C | A | rs11058647 | LINC02347 | Upstr | 1.31E-07 | **2.76E-07** | -0.09 | 0.494 |
| 12 | 126953511 | G | A | rs4765395 | LINC02347 | ncRNA | 1.45E-07 | **3.05E-07** | -0.09 | 0.409 |
| 12 | 126951336 | T | G | rs1029783 | LINC02347 | Intron | 1.47E-07 | **3.09E-07** | -0.09 | 0.470 |
| 12 | 126918017 | G | T | rs4765086 | LINC02347 | Upstr | 1.51E-07 | **3.18E-07** | -0.09 | 0.493 |
| 12 | 126951500 | G | A | rs7132331 | LINC02347 | Intron | 1.58E-07 | **3.32E-07** | -0.09 | 0.469 |
| 12 | 126924281 | T | C | rs11058645 | LINC02347 | Upstr | 1.72E-07 | **3.63E-07** | -0.09 | 0.495 |
| 12 | 126931379 | A | G | rs7485464 | LINC02347 | Intron | 1.73E-07 | **3.63E-07** | -0.09 | 0.493 |
| 12 | 126958047 | C | G | rs10847166 | LINC02347 | Downstr | 1.73E-07 | **3.64E-07** | -0.09 | 0.413 |
| 12 | 126921171 | T | G | rs722704 | LINC02347 | Upstr | 1.76E-07 | **3.71E-07** | -0.09 | 0.492 |
| 12 | 126951455 | T | C | rs2109149 | LINC02347 | Intron | 1.91E-07 | **4.01E-07** | -0.09 | 0.469 |
| 12 | 126955992 | C | T | rs10773282 | LINC02347 | Intron | 2.05E-07 | **4.32E-07** | -0.08 | 0.411 |
| 12 | 126951767 | C | A | rs1544542 | LINC02347 | Intron | 2.07E-07 | **4.35E-07** | -0.09 | 0.472 |
| 12 | 126936687 | C | T | rs4765386 | LINC02347 | Intron | 2.12E-07 | **4.45E-07** | -0.09 | 0.486 |
| 12 | 126932040 | G | A | rs2347023 | LINC02347 | Intron | 2.17E-07 | **4.57E-07** | -0.08 | 0.494 |
| 12 | 126932302 | A | G | rs2159387 | LINC02347 | Intron | 2.30E-07 | **4.83E-07** | -0.08 | 0.493 |
| 12 | 126932624 | G | A | rs758564 | LINC02347 | Intron | 2.54E-07 | 5.35E-07 | -0.08 | 0.493 |
| 12 | 126953471 | A | G | rs4765394 | LINC02347 | ncRNA | 2.97E-07 | 6.24E-07 | -0.08 | 0.410 |
| 12 | 126936552 | C | T | rs4765384 | LINC02347 | Intron | 3.19E-07 | 6.71E-07 | -0.08 | 0.485 |
| 12 | 126935088 | C | A | rs10773278 | LINC02347 | Intron | 3.54E-07 | 7.44E-07 | -0.08 | 0.486 |
| 12 | 126935280 | C | T | rs10773280 | LINC02347 | Intron | 3.54E-07 | 7.46E-07 | -0.08 | 0.485 |
| 12 | 126944767 | T | A | rs12296176 | LINC02347 | Intron | 3.60E-07 | 7.57E-07 | -0.08 | 0.484 |
| 12 | 126933597 | G | C | rs4765383 | LINC02347 | Intron | 3.66E-07 | 7.71E-07 | -0.08 | 0.485 |
| 12 | 126935114 | G | A | rs10773279 | LINC02347 | Intron | 3.70E-07 | 7.78E-07 | -0.08 | 0.485 |
| 12 | 126934371 | G | A | rs6489086 | LINC02347 | Intron | 3.79E-07 | 7.97E-07 | -0.08 | 0.485 |
| 12 | 126921766 | C | T | rs2109145 | LINC02347 | Upstr | 3.82E-07 | 8.05E-07 | -0.08 | 0.493 |
| 12 | 126934207 | G | A | rs7134778 | LINC02347 | Intron | 4.16E-07 | 8.76E-07 | -0.08 | 0.485 |
| 12 | 126923535 | G | T | rs2215304 | LINC02347 | Upstr | 4.34E-07 | 9.13E-07 | -0.08 | 0.487 |
| 12 | 126955454 | T | A | rs10492048 | LINC02347 | Intron | 4.36E-07 | 9.18E-07 | -0.08 | 0.410 |
| 12 | 126934892 | T | C | rs10847156 | LINC02347 | Intron | 4.40E-07 | 9.27E-07 | -0.08 | 0.485 |
| 12 | 126934220 | C | T | - | LINC02347 | Intron | 4.54E-07 | 9.56E-07 | -0.08 | 0.484 |
| 12 | 126934166 | G | A | rs7134668 | LINC02347 | Intron | 4.99E-07 | 1.05E-06 | -0.08 | 0.485 |

^1^ Nominal *p*-value.

^2^ Corrected for the effective number of independent traits tested: *p*-value^1^x*m_eff_* , and *m_eff_* = 2.105 for EA. Bold font: genome-wide suggestive significance (corrected *p*<5E-7).

## Table S7. Comprehensive list of loci and relevant functions associated with the AUD traits in AI and/or EA

All loci associated with any of the three studied AUD-related traits in either the American Indian (AI) or the European American (EA) cohort at an above genome-wide suggestively significant level. Bold font indicates genome-wide significance.

| **Genes** | **SNPs^a^** | **Trait –Test^b^** | ***P*-value^c^** | **Top Tissue-Specific Expressions (GTEx) ^d^** | **Relevant Functions** | **Previously Associated Related Traits or Disorders** |
| --- | --- | --- | --- | --- | --- | --- |
| **American Indian (AI)** | | | | | | |
| *NAF1-FSTL5* | **rs200577368** | 0-G | **6.35E-09** | Tibial Artery, Testis, lympohocytes | SNP located in a region of ~30bp that was methylated in frontal cortex, suggesting potential regulatory function^4^. See also *FSTL5.* |  |
| *DMRTA1* | rs79833306 | 0-G | 5.14E-08 | Liver,  Minor salivary gland, Esophagus:Mucosa |  | Expression level significantly elevated at ~3-fold change in blood during acute alcohol exposure (Expression Atlas ^5^) |
| *ANK1* | 4:41731811 | 0-G | 1.05E-07 | Cerebellar Hemisphere, Cerebelium,  Skeletal Muscle, Brain:Substantia nigra |  | Alzheimer’s ^6^, asthma ^7^, type II diabetes ^8^ |
| *ZIC2-PCCA* | 13:100709891 | 0-G | 1.10E-07 | *PCCA*: Adrenal Gland, Kidney Cortex, Cerebellar Hemisphere, Liver; *ZIC2*: Cerebellar Hemishpere, Cerebellium, Cortex | Protein encoded by *ZIC2* functions as a transcriptional repressor and may regulate tissue specific expression of dopamine receptor D1 | A histidine tract polymorphism in *ZIC2* might be associated with neural tube defects ^9^ |
| *BLZF1* | 1:169344460 | 0-G | 2.23E-07 | Testis, fibroblasts, Vagina |  |  |
| *KCNK2-KCTD3* | rs145751732 | 0-G | 2.48E-07 | Adrenal Gland,  Tibial Nerve,  Cerebella Hemisphere | Potassium channel genes. See *KCNK2* |  |
| *CR601322* | 2:114762469 | 0-G | 3.58E-07 |  |  |  |
| *MME (NEP,CD10)* | All variants (204)  Exon+Reg (36) | 0-F  0-R | 1.47E-05  5.35E-05 | fibroblasts,  Tibial Nerve,  Kidney Cortex | Encodes the highly conserved neutral endopeptidase (NEP) that cleaves and inactivates peptide hormones such as glucagon, enkephalins, substance P, neurotensin, oxytocin, and bradykinin ^10^ | Alzheimer’s disease ^11, 12^; alcohol consumption in both human and mice ^13, 14^ |
| ***KCNK2*** | Exon+Reg (11) | 0-R  1-R | **7.47E-07**  6.32E-06 | fibroblasts,  Adrenal Gland,  Tibial Nerve | Plays a key role in the cellular mechanisms of neuroprotection, anaesthesia, pain sensing and depression (see review by ^15^) | MDD and antidepressant treatment response ^16^ |
| *PRKG2* | **rs150351153** | 1-G | **9.75E-09** | Small Intestine: Terminal lieum, Esophagus:  Muscularis, Gastroesophageal Junction | Serine/threonine protein kinase family gene. Plays a key role in intestinal secretion by regulating cGMP-dependent translocation of CFTR in the jejunum. An inhibitor of *HCN2*, a potassium and sodium channel, in mouse ^17^. SNP located region methylated region in PFC. | Obesity traits in various ethnic groups ^18, 19^ |
| *DNAJB6* | rs139621545 | 1-G | 9.69E-08 | Skeletal Muscle,  Tibial Artery, Aorta |  | Candidate gene for ADHD ^20^; BMI ^21^ |
| *WDR75* | rs140545486 | 1-G | 1.03E-07 | lymphocytes, Ovary, Uterus |  | Ion status biomarker ^22^ |
| *DQ578965* | rs4099387 | 1-G | 1.24E-07 |  |  |  |
| *AK123120* | rs72769625 | 1-G | 1.90E-07 |  |  |  |
| *C4orf32* | rs76397899 | 1-G | 2.95E-07 | Adipose:  Visceral (Omentum), Subcutaneous, Mammary Tissue |  |  |
| *RREB1* | rs115143840 | 1-G | 3.64E-07 | Esophagus:Mucosa, Skin:Sun Exposed, Thyroid | Encodes a zinc finger transcription factor that may increase expression of calcitonin | Eotaxin level ^23^, adipocyte development in multi-ethnic populations ^24^, and substance-induced psychosis ^25^ |
| *C1orf99* | rs114401008 | 1-G | 4.91E-07 |  |  |  |
| *PARP15* | rs183811860 | 1-G | 5.03E-07 | Spleen, lymphocytes |  | MDD in European populations ^26^ |
| ***EBI3*** | Exon+Reg (7) | 1-R | **1.67E-07** | lymphocytes, Spleen, Small Intstine:  Terminal lieum | Subunit of the composite cytokines IL-27 and IL-35. Functions in innate immunity |  |
| *DSG1* | Nonsyn (15) | 1-R | 1.15E-05 | Skin: Sun Exposed, Not Sun Exposed,  Vagina | Encodes a cadherin-like transmembrane glycoprotein, and was identified as a target of auto-antibodies in an autoimmune skin disease | Eosinophilic esophagitis, an allergic inflammatory condition ^27^ |
| *FBXO45* | 3:196296922 | 2-G | 8.56E-08 | Cerebellar Hemisphere, Cerebelium, Testis |  |  |
| *AK310526* | rs75893595 | 2-G | 8.68E-08 |  |  |  |
| *BC040875* | 15:95883433 | 2-G | 1.46E-07 |  |  |  |
| *DPP10* | rs10189544 | 2-G | 1.56E-07 | Frontal Cortex(BA9), Cortex, Anterior cingulate cortex(BA24), Adrenal gland | Facilitates Kv4 channel surface expression and neuronal excitability | Asthma ^28^, obesity traits, bipolar disorders ^29^, response to antidepressants in depression ^30^, and paliperidone response in schizophrenia ^31^ |
| *PCLO* | rs144506483 | 2-G | 2.15E-07 | Cerebellar Hemiphere, Cerebelium, Pituitary |  | MDD ^32^, schizophrenia ^33^ [verify] |
| *BC038465* | 14:97964780 | 2-G | 3.42E-07 |  |  |  |
| *KIF2B* | 17:52323367 | 2-G | 3.45E-07 | Testis, Ectocervix |  |  |
| *LOC338797* | rs117113518 | 2-G | 3.45E-07 |  |  |  |
| *DFNB31* | 9:117198179 | 2-G | 4.34E-07 | Adrenal Gland, Testis, Pituitary |  | Depressive episodes in bipolar disorder ^34^ |
| *RP11-94B19.6* | Exon+Reg (7) | 2-R | 5.81E-06 | Testis, Ovary | Long non-coding RNA |  |
| *TICAM1* **^e^** | Exon+Reg (9) | 2-R | 1.57E-05 | Esophagus: Mucosa, lymphocytes,  Aorta Artery | Encodes a Toll/IL-1 like receptor adaptor protein and is involved in native immunity | Autoimmune vitiligo ^35^ |
| *ZNF644* **^e^** | Nonsyn (14) | 2-R | 1.85E-05 | Tibial Nerve, Endocervis, Ovary |  | Cognitive function ^36^, C-reactive protein and lipids ^37^, and metabolic syndrome ^38^ |
| **European American (EA/SFFS)** | | | | | | |
| *FSTL5* | rs11100375 | 0-G | 4.82E-07 | Cerebellar Hemisphere, Cerebelium, Hypothalamus | Calcium ion binding and protein binding | Alcoholism ^39^, alcohol dependence ^40^, response to amphetamines ^41^, interferon-γ induced monokine ^23^ and paliperidone response in schizophrenia ^31^ |
| *PDE4C* | Nonsyn | 0-R | 1.44E-06 | Transverse Colon, Tibial Artery,  Brain Cortex | Regulates the cellular concentration of cAMP. PDE4s act as pro-inflammatory enzymes through degradation of cAMP |  |
| *JARID2* | rs2500086 | 1-G | 2.83E-07 | Cerebellar Hemiphere, Cerebelium, Testis | Encodes a DNA-binding protein and functions as a transcriptional repressor |  |
| *IZUMO4* **^e^** | Nonsyn | 1-R | 1.34E-05 | Testis, lymphocytes |  |  |
| *LINC02347* | rs4309206 | 2-G | 9.08E-08 |  | See below: *LINC02347* |  |
| ***LINC02347***  ***/LOC100128554*** | All variants (155) | 2-F | **1.77E-07** | Testis, Frontal Cortex, Pancreas  (RP5-944M2.3 in GTEx) | Long non-coding RNA | MDD in European populations ^42^ |
| *COX19* **^e^** | Exon+Reg | 2-R | 1.37E-05 | Adrenal Gland,  Tibial Nerve,  Cerebellar Hemisphere | Encodes a cytochrome c oxidase (COX) assembly protein. Alcohol intoxication and withdrawal are known to generate oxidative stress that primarily target brain mitochondria | Oxidative stress decreases COX activity in a number of neurodegenerative diseases ^43^. Drug Topiramate for treating methamphetamine dependence alters *COX19* expression in the treatment responders ^44^ |

**^a^** Top SNP in the gene that has the highest significance in GWAS. Where dbSNP id is unavailable, chrom:position in reference genome hg19 is used. For rare-variant gene based test, Exon+Reg: variants on exons, 3’,5’UTR, upstream or downstream; Nonsyn: nonsynonymous or splice-site variants.

**^b^** Trait-Test pair: Trait 0: alcohol-related life events, 1: alcohol-induced affective symptoms during withdrawal, 2: alcohol-induced depression; Statistical Test: G: GWAS, F: FBAT gene-based test, R: rare-variant gene-based test.

**^c^** *P*-values are corrected for the number of traits. Bold font indicates genome-wide significance with respect to the test.

**^d^** Tissues are listed in the decreasing order of the median gene expression level. Only the top 2-4 tissues are listed.

**^e^** These loci have not passed the suggestive significant threshold after multi-test correction in the rare-variant gene-based test. They are included for potential relevance.

## Table S8. Top functional groups in AI

Functional groups from the top genes (*p*<1e-5, MAF≥1%) associated with alcohol-related life events and alcohol-induced affective symptoms in AI.

| Function | FDR | Genes in network | Genes in genome |
| --- | --- | --- | --- |
| **Alcohol-related life events (63 genes – 16)** ^1^ | | | |
| potassium ion transport | 1.46E-02 | 6 | 113 |
| potassium channel activity | 5.32E-02 | 5 | 91 |
| potassium ion transmembrane transporter activity | 5.32E-02 | 5 | 102 |
| monovalent inorganic cation transport | 5.32E-02 | 7 | 273 |
| oxidoreductase activity, acting on the aldehyde or oxo group of donors, NAD or NADP as acceptor | 1.59E-01 | 3 | 24 |
| metal ion transmembrane transporter activity | 1.74E-01 | 6 | 238 |
| oxidoreductase activity, acting on the aldehyde or oxo group of donors | 2.41E-01 | 3 | 32 |
| inorganic cation transmembrane transporter activity | 2.41E-01 | 6 | 266 |
| monovalent inorganic cation transmembrane transporter activity | 3.24E-01 | 5 | 180 |
| cation channel activity | 4.44E-01 | 5 | 197 |
| **Affective symptoms when cutting down or during withdrawal (44 genes – 12)** | | | |
| arachidonic acid metabolic process | 6.82E-02 | 4 | 51 |
| icosanoid metabolic process | 8.00E-02 | 4 | 70 |
| long-chain fatty acid metabolic process | 8.00E-02 | 4 | 75 |
| fatty acid derivative metabolic process | 8.00E-02 | 4 | 70 |
| unsaturated fatty acid metabolic process | 9.12E-02 | 4 | 82 |
| drug catabolic process | 5.91E-01 | 2 | 10 |
| epoxygenase P450 pathway | 6.19E-01 | 2 | 11 |
| **24 hours of depression when drinking (51 genes – 10)** | | | |
| None |  |  |  |

^1^ Number of genes with variant(s) having *p*<1e-5 in the GWAS minus the number of such gene symbols unrecognized by GeneMANIA.

## Table S9. Top functional groups in EA

Functional groups from the top genes (*p*<1e-5, MAF≥1%) associated with alcohol-related life events and dark side traits in SFFS.

| Function | FDR | Genes in network | Genes in genome |
| --- | --- | --- | --- |
| **Alcohol-related life events (40 genes – 5)** ^1^ | | | |
| regulation of Rac protein signal transduction | 1.10E-01 | 3 | 24 |
| regulation of Rac GTPase activity | 1.10E-01 | 3 | 21 |
| protein tyrosine kinase activity | 1.63E-01 | 4 | 98 |
| regulation of Rho GTPase activity | 1.63E-01 | 4 | 100 |
| regulation of Rho protein signal transduction | 2.75E-01 | 4 | 121 |
| transmembrane receptor protein tyrosine kinase activity | 3.44E-01 | 3 | 50 |
| regulation of Ras GTPase activity | 6.42E-01 | 4 | 175 |
| transmembrane receptor protein kinase activity | 6.42E-01 | 3 | 66 |
| ephrin receptor activity | 6.42E-01 | 2 | 14 |
| regulation of Ras protein signal transduction | 7.97E-01 | 4 | 213 |
| **Affective symptoms when cutting down or during withdrawal (40 genes – 13)** | | | |
| response to virus | 2.74E-05 | 8 | 205 |
| cellular response to type I interferon | 7.70E-04 | 5 | 74 |
| type I interferon signaling pathway | 7.70E-04 | 5 | 74 |
| response to type I interferon | 7.70E-04 | 5 | 75 |
| negative regulation of viral genome replication | 1.86E-03 | 4 | 38 |
| double-stranded RNA binding | 3.10E-03 | 4 | 45 |
| regulation of viral genome replication | 4.42E-03 | 4 | 51 |
| negative regulation of viral process | 8.24E-03 | 4 | 65 |
| viral genome replication | 8.24E-03 | 4 | 64 |
| adenylyltransferase activity | 8.24E-03 | 3 | 18 |
| negative regulation of multi-organism process | 1.64E-02 | 4 | 79 |
| regulation of multi-organism process | 4.70E-02 | 5 | 216 |
| defense response to virus | 6.31E-02 | 4 | 116 |
| **24 hours of depression when drinking (31 genes – 5)** | | | |
| None |  |  |  |

^1^ Number of genes with variant(s) having *p*<1e-5 in the GWAS minus the number of such gene symbols unrecognized by GeneMANIA.

## Table S10. Disease enrichment in AI

Enriched diseases from the top genes (*p*<1e-5, MAF≥1%) associated with alcohol-related life events and alcohol-induced affective symptoms in AI.

| Disease^1^ | Count | %Genes | *p*-value | *p*-value^2^ | Genes |
| --- | --- | --- | --- | --- | --- |
| **Alcohol-related life events** | | | | | |
| CHEMDEPENDENCY | 18 | 39.1 | 0.015 | 0.229 | PTPRD, OSBP2, RGS7BP, LRRC4C, CDH4, KCNK2, DMRTA1, ELL2, NPAS2, DHRS3, ANK1, KCNK9, FSIP1, SORBS2, SYN3, DAP, PCCA, ALDH9A1 |
| Insulin | 5 | 10.9 | 0.002 | 0.285 | PTPRD, ANK1, SYN3, LRRC4C, ELL2 |
| CARDIOVASCULAR | 19 | 41.3 | 0.033 | 0.439 | PTPRD, OSBP2, MME, LRRC4C, TTN, ANKRD55, KCNK2, CDH4, DMRTA1, ELL2, RNF130, BLZF1, HMCN1, ANK1, FSIP1, CXCR4, SYN3, PCCA, ALDH9A1 |
| **Affective symptoms when cutting down or during withdrawal** | | | | | |
| CHEMDEPENDENCY | 14 | 45.2 | 0.001 | **0.021** | PHACTR1, PLXNA4, CYP2C8, PLD5, PPFIBP2, CACNB2, PRKG2, COL5A2, PCDHGA1, RNF180, PLA2G4A, GRM6, GALNTL6, NFATC2 |
| Tobacco Use Disorder | 14 | 45.2 | 0.000 | **0.028** | PHACTR1, PLXNA4, CYP2C8, PLD5, PPFIBP2, CACNB2, PRKG2, COL5A2, PCDHGA1, RNF180, PLA2G4A, GRM6, GALNTL6, NFATC2 |
| **24 hours of depression when drinking** | | | | | |
| CARDIOVASCULAR | 20 | 52.6 | 0.001 | **0.018** | DCBLD2, CNTN5, COL13A1, PTPRN2, PREX1, SNX16, FASLG, LRRC4C, ANKRD55, FAM13A, DMRTA1, LHFPL3, HMCN1, IMPG1, P2RY2, ANKRD11, EBF1, PPP2R5E, RAPGEF2, PCCA |
| Lipoproteins, VLDL | 5 | 13.2 | 0.001 | 0.097 | SPG7, FASLG, LRRC4C, RAPGEF2, DMRTA1 |
| HEMATOLOGICAL | 10 | 26.3 | 0.008 | 0.123 | DCBLD2, SPG7, CNTN5, IMPG1, EBF1, FASLG, LRRC4C, RAPGEF2, DMRTA1, PCCA |
| CHEMDEPENDENCY | 16 | 42.1 | 0.013 | 0.196 | ADCY3, DCBLD2, ENAH, CNTN5, PTPRN2, PREX1, FASLG, LRRC4C, FAM13A, DMRTA1, LHFPL3, SORBS2, IMPG1, EBF1, DSC3, PCCA |
| Hemoglobins | 5 | 13.2 | 0.003 | 0.466 | DCBLD2, IMPG1, RAPGEF2, DMRTA1, PCCA |

^1^ Disease class (all-caps) or disease (start case) from genetic association database (GAD)

^2^ Bonferroni corrected *p*-value

## Table S11. Disease enrichment in EA

Enriched diseases from the top genes (*p*<1e-5, MAF≥1%) associated with alcohol-related life events and alcohol-induce affective symptoms in SFFS.

| Disease^1^ | Count | %Genes | *p*-value | *p*-value^2^ | Genes |
| --- | --- | --- | --- | --- | --- |
| **Alcohol-related life events** | | | | | |
| PSYCH | 7 | 24.1 | 0.044 | 0.471 | RNF150, EPHA6, SLC12A2, GNAI1, OSBPL6, DGKI, PTBP2 |
| **Affective symptoms when cutting down or during withdrawal** | | | | | |
| CHEMDEPENDENCY | 14 | 56.0 | 0.004 | 0.063 | NDST4, FAM110B, OAS3, DGKH, EML6, CHD9, APOB, ST6GALNAC3, RNF150, SORBS2, SIAH3, GFRA1, DENND3, GNG7 |
| Tobacco Use Disorder | 13 | 52.0 | 0.003 | 0.471 | CHD9, ST6GALNAC3, APOB, RNF150, NDST4, SORBS2, FAM110B, SIAH3, GFRA1, DGKH, DENND3, EML6, GNG7 |
| **24 hours of depression when drinking** | | | | | |
| CHEMDEPENDENCY | 11 | 47.8 | 0.013 | 0.208 | MBL2, ABLIM2, SAMD12, XIRP2, HS3ST2, FSTL5, KIAA1549, CNTNAP2, PLCB1, LRFN5, OSCP1 |

^1^ Disease class (all-caps) or disease (start case) from genetic association database (GAD)

^2^ Bonferroni corrected *p*-value

## Table S12. Tissue-specific gene expression enrichment in AI

Tissues with enriched gene expression from the top genes (*p*<1e-5, MAF≥1%) associated with alcohol-related life events and alcohol-induce affective symptoms in AI.

|  | #Genes | *p*-value^1^ |
| --- | --- | --- |
| **Alcohol-related life events, 46 (63)** ^2^ | | |
| Adrenal Gland | 5 | 0.001 |
| Adipose - Visceral (Omentum) | 4 | 0.003 |
| Breast - Mammary Tissue | 2 | 0.036 |
| Artery - Tibial | 4 | 0.041 |
| Cells - Transformed fibroblasts | 6 | 0.056 |
| Brain - Nucleus accumbens (basal ganglia) | 3 | 0.085 |
| Brain - Cerebellar Hemisphere | 10 | 0.090 |
| Minor Salivary Gland | 3 | 0.097 |
| **Affective symptoms when cutting down or during withdrawal, 31 (44)** | | |
| Esophagus - Gastroesophageal Junction | 2 | 0.013 |
| Esophagus - Muscularis | 2 | 0.027 |
| Brain - Nucleus accumbens (basal ganglia) | 3 | 0.030 |
| Cells - Transformed fibroblasts | 5 | 0.039 |
| Brain - Frontal Cortex (BA9) | 4 | 0.043 |
| Brain - Cerebellar Hemisphere | 8 | 0.051 |
| Adipose - Visceral (Omentum) | 2 | 0.052 |
| Brain - Anterior cingulate cortex (BA24) | 2 | 0.055 |
| **24 hours of depression when drinking, 39 (51)** | | |
| Brain - Nucleus accumbens (basal ganglia) | 5 | 0.001 |
| Artery - Tibial | 5 | 0.005 |
| Adipose - Subcutaneous | 3 | 0.028 |
| Brain - Caudate (basal ganglia | 2 | 0.070 |
| Brain - Anterior cingulate cortex (BA24) | 2 | 0.071 |
| Brain - Frontal Cortex (BA9) | 4 | 0.073 |
| Brain - Hypothalamus | 2 | 0.075 |
| Brain - Cortex | 3 | 0.093 |
| Esophagus - Mucosa | 3 | 0.093 |

^1^ Determined through 1000 permutations

^2^ Number of gene symbols recognized by GTEx (number of total top gene symbols)

## Table S13. Tissue-specific gene expression enrichment in EA

Tissues with enriched gene expression from top genes (*p*<1e-5, MAF≥1%) associated with alcohol-related life events and alcohol-induced affective symptoms in SFFS.

|  | #Genes | *p*-value^1^ |
| --- | --- | --- |
| **Alcohol-related life events 28 (40)** ^2^ | | |
| Brain - Cortex | 5 | 0.001 |
| Brain - Frontal Cortex (BA9) | 6 | 0.001 |
| Brain - Anterior cingulate cortex (BA24) | 3 | 0.009 |
| Skin - Sun Exposed (Lower leg | 4 | 0.021 |
| Brain - Nucleus accumbens (basal ganglia) | 3 | 0.025 |
| Brain - Spinal cord (cervical c 1) | 3 | 0.052 |
| Colon - Sigmoid | 2 | 0.064 |
| Thyroid | 4 | 0.085 |
| **Affective symptoms when cutting down or during withdrawal 25 (41)** | | |
| Colon - Sigmoid | 3 | 0.005 |
| Artery - Tibial | 3 | 0.023 |
| Adipose - Visceral (Omentum) | 2 | 0.025 |
| Fallopian Tube | 3 | 0.026 |
| Heart - Atrial Appendage | 2 | 0.029 |
| **24 hours of depression when drinking 23 (31)** | | |
| Brain - Frontal Cortex (BA9) | 7 | 0.001 |
| Brain - Cortex | 5 | 0.002 |
| Muscle - Skeletal | 4 | 0.003 |
| Brain - Caudate (basal ganglia) | 2 | 0.049 |
| Brain - Cerebellar Hemisphere | 6 | 0.064 |

^1^ Determined through 1000 permutations

^2^ Number of gene symbols recognized by GTEx (number of total top gene symbols)

# Supplemental Materials and Methods

## Participants

*American Indian Cohort (AI).* Nine hundred and three (903) Native Americans from extended pedigrees participated in the study. The population characteristics and the recruitment procedures have been previously described ^45^. Participants who had at least one-sixteenth self-reported American-Indian heritage were targeted and recruited for the study as previously described ^46^. Seven hundred-fifty (750) individuals had their whole genome sequenced.

*San Francisco Family Alcohol Study (SFFS).* The SFFS was a nationwide genetic study on alcoholism. The recruitment process and the population characteristics have been previous described ^47-49^. Over 90% are European Americans. 2524 individuals participated in the study. Eighteen hundred and eighty-nine (1889) participants had their whole genome sequenced.

All participants were assessed using the Semi-Structured Assessment for the Genetics of Alcoholism (SSAGA) ^50-52^ in order to collect demographic information and to make DSM-IV diagnoses ^53^. An individual was removed if missing all three phenotypes in the present study; 742 participants from the American Indian cohort and 1711 participants from the SFFS cohort remained for further analysis. Their demographic data is listed in Table S1.

## Whole genome sequencing and variants calling

The same methods and pipeline were used to sequence the AI and the SFFS cohorts, and has been previously published ^54^. In brief, blood derived DNA was sequenced using Illumina low-coverage whole genome sequencing (LCWGS), as well as genotyped using an Affymetrix Exome1A chip. The pair-end sequencing was performed on HiSeq2000 sequencers (Illumina). About 80% of the samples had coverage between 3X and 12X, approximately evenly distributed. Reads from whole genome sequencing were aligned to the GRCh37/hg19 human reference genome using BMA, and realigned near indels with GATK ^55^. Variants were called using both GATK Unified Genotyper following the best practices for low-coverage samples ^56^ and the LD-aware variant caller Thunder ^57^. Imputation was carried out using the program Thunder. Qualities of variant calling were assessed through a comparison between the sequencing results to genotypes generated on the exome array for the same set of subjects. The assessment showed nearly all of common variants and a high percentage of rare variants in the samples were correctly called. The median concordance rate was 97.5%. The median false positive rate was 0.3% ^54^. The variants for each cohort were called separately, resulting in 23,550,342 genome-wide variants for 750 AI individuals, and 33,752,749 variants for 1889 SFFS individuals.

## Whole genome association analysis

Most participants in the American Indian cohort have admixed ancestral background, both indicated by self-report and by admixture analysis ^58, 59^. While SFFS cohort is primarily composed of European American, small percentages of the participants are from different ethnic groups (Table S1). In addition, both cohorts have family structures: AI has large extended pedigrees; SFFS is made up of small families. Rather than removing samples either due to relatedness or different populations followed by correcting the broad sample structure using principle components, we used a linear mixed model approach as implemented in EMMAX ^60^ in the association analysis to control for both population structures and familial relatedness. Prior to the association analysis, a kinship matrix was estimated from genotypes to capture a wide range of sample structures. The association was then conditioned on the estimated kinship matrix. We further included sex, age and age-squared as covariates in all association analyses. For the three AUD traits, the genomic control (*λ*_GC_) values ranged from 0.998 to 1.009 for the AI cohort and from 1.024 to 1.079 for the SFFS cohort, indicating that the population stratification or relatedness had been largely controlled for and there was no inflation or deflation due to these factors. See Figure S1 for the Q-Q plots of all GWAS.

We further corrected the significant values of associations for multiple traits. Since the traits were correlated (Table S4), we corrected for the effective independent number of traits (see below) ^61^. We used *p*-value of 5x10^-8^ as the genome-wide significant threshold and 5x10^-7^ as the threshold for suggestive significance.

Additionally, we performed a gene-based test using fastBAT ^62^. For each gene, all variants in the range of ±50 Kb of the gene and of MAF≥1% were included. SNPs that are in high LD (*r*^2^ > 0.9) with each other were pruned. The *p*-values were corrected for the effective number of traits. The number of genes (*N*) was 24690 and 24681 for AI and SFFS respectively. Thus the significant threshold for *p*-value using Bonferroni correction was set at 0.05/*N* = 2.0 x 10^-6^, and suggestively significant threshold at 2.0 x 10^-5^.

## Effective number of independent phenotypes

It has been shown that the total amount of correlation among a set of variables can be measured by the variance of the eigenvalues (λ_s_) derived from the correlation matrix ^61, 63^. Higher correlation among variables leads to higher variance of λ_s_. Let *M* be the number of variables represented in the correlation matrix, then the variance of λ_s_ will range between zero, when all variables are independent, and *M*, when collective correlation is at its maximum. The proportional reduction of the number of variables can therefore be estimated by the ratio of the observed eigenvalue variance (Var(λ)) to its maximum value (*M*). The effective number of independent variables can then be calculated as

$$M_{eff}=1+(M-1)\left( 1-\frac{Var\left( \lambda\right)}{M} \right)$$

To obtain the effective number of independent traits, we first residualized the AUD phenotypes over the covariates including sex, age and age-squared. We then computed the observed variance of the eigenvalues of the correlation matrix of the residuals. For the three traits in the present study, *m_eff_* = 2.369 and 2.105 for the AI and SFFS cohorts respectively.

## Gene-based low-frequency and rare variants association analysis

A liner mixed model based combined multivariate and collapsing method ^64^ as implemented in EMMAX was used to analyze the variants having less than 5% minor allele frequency (low-frequency variants: 1% ≤ MAF < 5%, and rare variants: MAF < 1%). We grouped the low-frequency and rare variants by genes. For each gene, we formed two types of groups. One group considered all variants on exons, 5’ and 3’ UTRs, upstream and downstream of the gene. The other group included only the nonsynonymous variants and the splicing-site variants of the gene. Intergenic variants were not considered in the present study. For each group type, a gene was excluded if fewer than three markers were found, or if less than 1% of the samples had any such markers on the gene. The gene-based burden test was performed on the alcohol-related life events and the alcohol-induced affective symptoms phenotypes. The *p*-values were corrected for *m_eff_*. The significant thresholds for corrected *p*-values were set at 0.05/(*N_Exon+Reg_* + *N_Nonsyn_*), where *N_Exon+Reg_* is the number of genes in the group Exon+Reg and *N_Nonsyn_* in the group Nonsyn, for each trait and cohort. Note that correcting for the sum of the numbers of genes in two groups is likely an overcorrection as two groups of variants are correlated.

## Functional and pathway analyses

Brain-specific *cis*-eQTL was obtained from BRAINEAC ^65^ dataset. The dataset consists of genotypes and gene expressions of ten post-mortem brain tissues from each of the 134 individuals of European descent, and the eQTL signals were assessed at the exon level ^66^. Top variants from our association analyses were tested against this database. For nonsynonymous variants, Polyphen-2 was used to predict whether the variants might be potentially damaging ^67^.

The variants with *p*-value < 10^-5^ from each GWAS were first annotated with SGAdviser ^68^. Each variant was assigned a gene either on which the variant resided or to which the variant was the closest. The resulting sets of genes were then subjected to functional analyses. We used GeneMANIA ^69^ to extract potential functional networks associated with each gene set, along with an additional number of related genes selected by GeneMANIA (the additional number of genes to include was 25% of the number of genes in the gene set recognized by the software). The following networks were included in the analysis: co-expression, co-localization, and consolidated pathways. We further used DAVID 6.8 ^70^ for a disease enrichment analysis based on the genetic association database (GAD). We report their Bonferroni corrected *p*-values.

## Tissue-specific gene expression enrichment analysis

We obtained the median tissue-specific gene expression data (in TPMs: Transcripts Per Kb Million) from The Genotype-Tissue Expression (GTEx) Project release V7 (at GTEx Portal) for the sets of genes associated with variants that had *p*-value < 10^-5^ in the GWAS of each trait and cohort. For each gene, its expression profile across tissues was first standardized. For each tissue *T*, we then counted the number of genes in each gene set that had expression levels over z-score of 2 (for any such gene, *T* was considered among the most expressed tissues by that gene) and denoted this expressed-gene-count as *T_g_*. If *T_g_* was significantly higher than expected, the tissue *T* was considered enriched with respect to tissue-specific expressions for the gene set. The significance was determined through permutation test as the following.

Let *n* be the number of genes in the gene set. At each iteration *i*, we randomly selected *n* genes from the GTEx dataset. We then obtained the most expressed tissues by each gene in this random gene set and from which derived the expressed-gene-count *T_g_^i^* for tissue *T*. The significance of enrichment of *T* was determined by |{*T_g_^i^* ≥ *T_g_* | *i=1,…,m*}|/(*m*+1), where *m* is the number of permutations. A thousand permutations (*m*=1000) were performed for each gene set, yielding the smallest possible *p*-value of 1/1001.

# Supplemental References

1. Purcell S, Cherny SS, Sham PC. Genetic Power Calculator: design of linkage and association genetic mapping studies of complex traits. *Bioinformatics* 2003; **19**(1)**:** 149-150.

2. Sham PC, Purcell SM. Statistical power and significance testing in large-scale genetic studies. *Nat Rev Genet* 2014; **15**(5)**:** 335-346.

3. Bůžková P. Linear Regression in Genetic Association Studies. *PLOS ONE* 2013; **8**(2)**:** e56976.

4. Maunakea AK*, et al*. Conserved role of intragenic DNA methylation in regulating alternative promoters. *Nature* 2010; **466:** 253.

5. Kapushesky M*, et al*. Gene Expression Atlas update—a value-added database of microarray and sequencing-based functional genomics experiments. *Nucleic Acids Research* 2012; **40**(D1)**:** D1077-D1081.

6. Mastroeni D*, et al*. ANK1 is up-regulated in laser captured microglia in Alzheimer’s brain; the importance of addressing cellular heterogeneity. *PLOS ONE* 2017; **12**(7)**:** e0177814.

7. Imboden M*, et al*. Genome-wide association study of lung function decline in adults with and without asthma. *The Journal of Allergy and Clinical Immunology* 2012; **129**(5)**:** 1218-1228.

8. Zhao W*, et al*. Identification of new susceptibility loci for type 2 diabetes and shared etiological pathways with coronary heart disease. *Nature Genetics* 2017; **49:** 1450.

9. Brown LY, Hodge SE, Johnson WG, Guy SG, Nye JS, Brown S. Possible association of NTDs with a polyhistidine tract polymorphism in the ZIC2 gene. *American Journal of Medical Genetics* 2002; **108**(2)**:** 128-131.

10. Turner AJ, Tanzawa K. Mammalian membrane metallopeptidases: NEP, ECE, KELL, and PEX. *The FASEB Journal* 1997; **11**(5)**:** 355-364.

11. Iwata N*, et al*. Metabolic Regulation of Brain Aβ by Neprilysin. *Science* 2001; **292**(5521)**:** 1550.

12. Miners JS, Van Helmond Z, Chalmers K, Wilcock G, Love S, Kehoe PG. Decreased Expression and Activity of Neprilysin in Alzheimer Disease Are Associated With Cerebral Amyloid Angiopathy. *Journal of Neuropathology & Experimental Neurology* 2006; **65**(10)**:** 1012-1021.

13. Frette C*, et al*. Relationship of Serum Neutral Endopeptidase E.C.3,4,24.11 Activity to Alcohol Consumption. *Alcoholism: Clinical and Experimental Research* 1998; **22**(7)**:** 1405-1408.

14. Siems W-E*, et al*. Neutral endopeptidase and alcohol consumption, experiments in neutral endopeptidase-deficient mice. *European Journal of Pharmacology* 2000; **397**(2)**:** 327-334.

15. Honore E. The neuronal background K2P channels: focus on TREK1. *Nat Rev Neurosci* 2007; **8**(4)**:** 251-261.

16. Liou YJ, Chen TJ, Tsai S-J, Yu YWY, Cheng C-Y, Hong C-J. Support for the involvement of the KCNK2 gene in major depressive disorder and response to antidepressant treatment. *Pharmacogenet Genomics* 2009; **19**(10)**:** 735-741.

17. Hammelmann V, Zong X, Hofmann F, Michalakis S, Biel M. The cGMP-Dependent Protein Kinase II Is an Inhibitory Modulator of the Hyperpolarization-Activated HCN2 Channel. *PLOS ONE* 2011; **6**(2)**:** e17078.

18. Graff M*, et al*. Genome-wide physical activity interactions in adiposity ― A meta-analysis of 200,452 adults. *PLOS Genetics* 2017; **13**(4)**:** e1006528.

19. Justice AE*, et al*. Genome-wide meta-analysis of 241,258 adults accounting for smoking behaviour identifies novel loci for obesity traits. *Nature Communications* 2017; **8:** 14977.

20. Lasky-Su J*, et al*. Genome-wide association scan of quantitative traits for attention deficit hyperactivity disorder identifies novel associations and confirms candidate gene associations. *American Journal of Medical Genetics Part B: Neuropsychiatric Genetics* 2008; **147B**(8)**:** 1345-1354.

21. Locke AE*, et al*. Genetic studies of body mass index yield new insights for obesity biology. *Nature* 2015; **518:** 197.

22. Benyamin B*, et al*. Novel loci affecting iron homeostasis and their effects in individuals at risk for hemochromatosis. *Nature Communications* 2014; **5:** 4926.

23. Ahola-Olli AV*, et al*. Genome-wide Association Study Identifies 27 Loci Influencing Concentrations of Circulating Cytokines and Growth Factors. *The American Journal of Human Genetics* 2016; **100**(1)**:** 40-50.

24. Chu AY*, et al*. Multiethnic genome-wide meta-analysis of ectopic fat depots identifies loci associated with adipocyte development and differentiation. *Nature Genetics* 2016; **49:** 125.

25. Ohadi M*, et al*. Novel evidence of the involvement of calreticulin in major psychiatric disorders. *Progress in Neuro-Psychopharmacology and Biological Psychiatry* 2012; **37**(2)**:** 276-281.

26. Major Depressive Disorder Working Group of the Psychiatric GC. A mega-analysis of genome-wide association studies for major depressive disorder. *Molecular Psychiatry* 2012; **18:** 497.

27. Rothenberg ME*, et al*. Common variants at 5q22 associate with pediatric eosinophilic esophagitis. *Nature Genetics* 2010; **42:** 289.

28. Gao J*, et al*. Polymorphisms of <i>PHF11</i> and <i>DPP10 </i> Are Associated with Asthma and Related Traits in a Chinese Population. *Respiration* 2010; **79**(1)**:** 17-24.

29. Djurovic S*, et al*. A genome-wide association study of bipolar disorder in Norwegian individuals, followed by replication in Icelandic sample. *Journal of Affective Disorders* 2010; **126**(1)**:** 312-316.

30. Li QS, Tian C, Seabrook GR, Drevets WC, Narayan VA. Analysis of 23andMe antidepressant efficacy survey data: implication of circadian rhythm and neuroplasticity in bupropion response. *Translational Psychiatry* 2016; **6:** e889.

31. Li Q*, et al*. Genome-wide association study of paliperidone efficacy. *Pharmacogenetics and Genomics* 2017; **27**(1)**:** 7-18.

32. Mbarek H*, et al*. Genome-Wide Significance for PCLO as a Gene for Major Depressive Disorder. *Twin Research and Human Genetics* 2017; **20**(4)**:** 267-270.

33. Goes FS*, et al*. Genome-wide association study of schizophrenia in Ashkenazi Jews. *American Journal of Medical Genetics Part B: Neuropsychiatric Genetics* 2015; **168**(8)**:** 649-659.

34. Fabbri C, Serretti A. Genetics of long-term treatment outcome in bipolar disorder. *Progress in Neuro-Psychopharmacology and Biological Psychiatry* 2016; **65:** 17-24.

35. Jin Y*, et al*. Genome-wide association studies of autoimmune vitiligo identify 23 new risk loci and highlight key pathways and regulatory variants. *Nature Genetics* 2016; **48:** 1418.

36. Trampush JW*, et al*. GWAS meta-analysis reveals novel loci and genetic correlates for general cognitive function: a report from the COGENT consortium. *Molecular Psychiatry* 2017; **22:** 336.

37. Ligthart S*, et al*. Bivariate genome-wide association study identifies novel pleiotropic loci for lipids and inflammation. *BMC Genomics* 2016; **17**(1)**:** 443.

38. Zabaneh D, Balding DJ. A Genome-Wide Association Study of the Metabolic Syndrome in Indian Asian Men. *PLOS ONE* 2010; **5**(8)**:** e11961.

39. Heath AC*, et al*. A Quantitative-Trait Genome-Wide Association Study of Alcoholism Risk in the Community: Findings and Implications. *Biological Psychiatry* 2011; **70**(6)**:** 513-518.

40. McGue M*, et al*. A Genome-Wide Association Study of Behavioral Disinhibition. *Behavior Genetics* 2013; **43**(5)**:** 363-373.

41. Hart AB*, et al*. Genome-Wide Association Study of d-Amphetamine Response in Healthy Volunteers Identifies Putative Associations, Including Cadherin 13 (CDH13). *PLOS ONE* 2012; **7**(8)**:** e42646.

42. Davies MN*, et al*. Hypermethylation in the ZBTB20 gene is associated with major depressive disorder. *Genome Biology* 2014; **15**(4)**:** R56.

43. Manzo-Avalos S, Saavedra-Molina A. Cellular and Mitochondrial Effects of Alcohol Consumption. *International Journal of Environmental Research and Public Health* 2010; **7**(12)**:** 4281-4304.

44. Niu T, Li J, Wang J, Ma JZ, Li MD. Identification of Novel Signal Transduction, Immune Function, and Oxidative Stress Genes and Pathways by Topiramate for Treatment of Methamphetamine Dependence Based on Secondary Outcomes. *Frontiers in Psychiatry* 2017; **8:** 271.

45. Ehlers CL, Gizer IR, Gilder DA, Wilhelmsen KC. Linkage analyses of stimulant dependence, craving, and heavy use in American Indians. *American Journal of Medical Genetics Part B: Neuropsychiatric Genetics* 2011; **156B**(7)**:** 772-780.

46. Ehlers CL, Spence JP, Wall TL, Gilder DA, Carr LG. Association of ALDH1 promoter polymorphisms with alcohol-related phenotypes in southwest California Indians. *Alcohol Clin Exp Res* 2004; **28**(10)**:** 1481-1486.

47. Seaton KL, Cornell JL, Wilhelmsen KC, Vieten C. Effective strategies for recruiting families ascertained through alcoholic probands. *Alcohol Clin Exp Res* 2004; **28**(1)**:** 78-84.

48. Vieten C, Seaton KL, Feiler HS, Wilhelmsen KC. The University of California, San Francisco Family Alcoholism Study. I. Design, Methods, and Demographics. *Alcoholism: Clinical and Experimental Research* 2004; **28**(10)**:** 1509-1516.

49. Gizer IR*, et al*. Linkage scan of alcohol dependence in the UCSF Family Alcoholism Study. *Drug and Alcohol Dependence* 2011; **113**(2–3)**:** 125-132.

50. Bucholz KK*, et al*. A new, semi-structured psychiatric interview for use in genetic linkage studies: a report on the reliability of the SSAGA. *Journal of Studies on Alcohol* 1994; **55**(2)**:** 149-158.

51. Hesselbrock M, Easton C, Bucholz KK, Schuckit M, Hesselbrock V. A validity study of the SSAGA--a comparison with the SCAN. *Addiction* 1999; **94**(9)**:** 1361-1370.

52. Wall TL, Carr LG, Ehlers CL. Protective association of genetic variation in alcohol dehydrogenase with alcohol dependence in Native American Mission Indians. *American Journal of Psychiatry* 2003; **160**(1)**:** 41-46.

53. American Psychiatric Association Task Force on DSM-IV. *Diagnostic and Statistical Manual of Mental Disorder (DSM-IV)*. American Psychiatric Association: Washington, DC, 1994.

54. Bizon C*, et al*. Variant calling in low-coverage whole genome sequencing of a Native American population sample. *BMC Genomics* 2014; **15**(1)**:** 85.

55. DePristo MA*, et al*. A framework for variation discovery and genotyping using next-generation DNA sequencing data. *Nature Genetics* 2011; **43**(5)**:** 491-498.

56. Van der Auwera GA*, et al*. From FastQ Data to High-Confidence Variant Calls: The Genome Analysis Toolkit Best Practices Pipeline. *Current Protocols in Bioinformatics*. John Wiley & Sons, Inc., 2002.

57. Li Y, Sidore C, Kang HM, Boehnke M, Abecasis GR. Low-coverage sequencing: implications for design of complex trait association studies. *Genome Research* 2011; **21**(6)**:** 940-951.

58. Norden-Krichmar TM, Gizer IR, Libiger O, Wilhelmsen KC, Ehlers CL, Schork NJ. Correlation analysis of genetic admixture and social identification with body mass index in a Native American community. *Am J Hum Biol* 2014; **26**(3)**:** 347-360.

59. Peng Q*, et al*. Association and ancestry analysis of sequence variants in ADH and ALDH using alcohol-related phenotypes in a Native American community sample. *American Journal of Medical Genetics Part B: Neuropsychiatric Genetics* 2014; **165**(8)**:** 673-683.

60. Kang HM*, et al*. Variance component model to account for sample structure in genome-wide association studies. *Nature Genetics* 2010; **42:** 348-354.

61. Nyholt DR. A simple correction for multiple testing for single-nucleotide polymorphisms in linkage disequilibrium with each other. *The American Journal of Human Genetics* 2004; **74**(4)**:** 765-769.

62. Bakshi A*, et al*. Fast set-based association analysis using summary data from GWAS identifies novel gene loci for human complex traits. *Scientific Reports* 2016; **6:** 32894.

63. Cheverud JM. A simple correction for multiple comparisons in interval mapping genome scans. *Heredity (Edinb)* 2001; **87**(Pt 1)**:** 52-58.

64. Li B, Leal SM. Methods for detecting associations with rare variants for common diseases: application to analysis of sequence data. *The American Journal of Human Genetics* 2008; **83**(3)**:** 311-321.

65. Ramasamy A*, et al*. Genetic variability in the regulation of gene expression in ten regions of the human brain. *Nat Neurosci* 2014; **17**(10)**:** 1418-1428.

66. Trabzuni D*, et al*. Quality control parameters on a large dataset of regionally dissected human control brains for whole genome expression studies. *Journal of Neurochemistry* 2011; **119**(2)**:** 275-282.

67. Adzhubei IA*, et al*. A method and server for predicting damaging missense mutations. *Nature methods* 2010; **7**(4)**:** 248-249.

68. Pham PH, Shipman WJ, Erikson GA, Schork NJ, Torkamani A. Scripps Genome ADVISER: Annotation and Distributed Variant Interpretation SERver. *PLoS One* 2015; **10**(2)**:** e0116815.

69. Warde-Farley D*, et al*. The GeneMANIA prediction server: biological network integration for gene prioritization and predicting gene function. *Nucleic Acids Research* 2010; **38**(suppl 2)**:** W214-W220.

70. Huang DW, Sherman BT, Lempicki RA. Systematic and integrative analysis of large gene lists using DAVID bioinformatics resources. *Nature Protocols* 2008; **4:** 44.
